# The dental plaque biofilm matrix

**DOI:** 10.1111/prd.12361

**Published:** 2021-03-10

**Authors:** Nicholas S. Jakubovics, Steven D. Goodman, Lauren Mashburn-Warren, Graham P. Stafford, Fabian Cieplik

**Affiliations:** 1 School of Dental Sciences, Newcastle University, Newcastle upon Tyne, UK; 2 Center for Microbial Pathogenesis, The Abigail Wexner Research Institute at Nationwide Children’s Hospital, The Ohio State University College of Medicine, Columbus, Ohio, USA; 3 Integrated Biosciences, School of Clinical Dentistry, University of Sheffield, Sheffield, UK; 4 Department of Conservative Dentistry and Periodontology, University Hospital Regensburg, Regensburg, Germany

## 1 | INTRODUCTION

Advances in deoxyribonucleic acid (DNA) sequencing have given us unprecedented insights into the identity of microbial cells within complex consortia, such as dental plaque biofilms. Our understanding of the other key component of microbial biofilms, the extracellular matrix, has not benefited to such an extent from this technological revolution. Nevertheless, a great deal of progress has been made in recent years toward defining the structure and function of biofilm matrices. The roles of macromolecules, such as nucleic acids, proteins, and lipids, in addition to polysaccharides, are beginning to be unraveled. A more thorough characterization of these components may lead to new approaches to control biofilms based on inhibition of matrix function.

**FIGURE 1:**
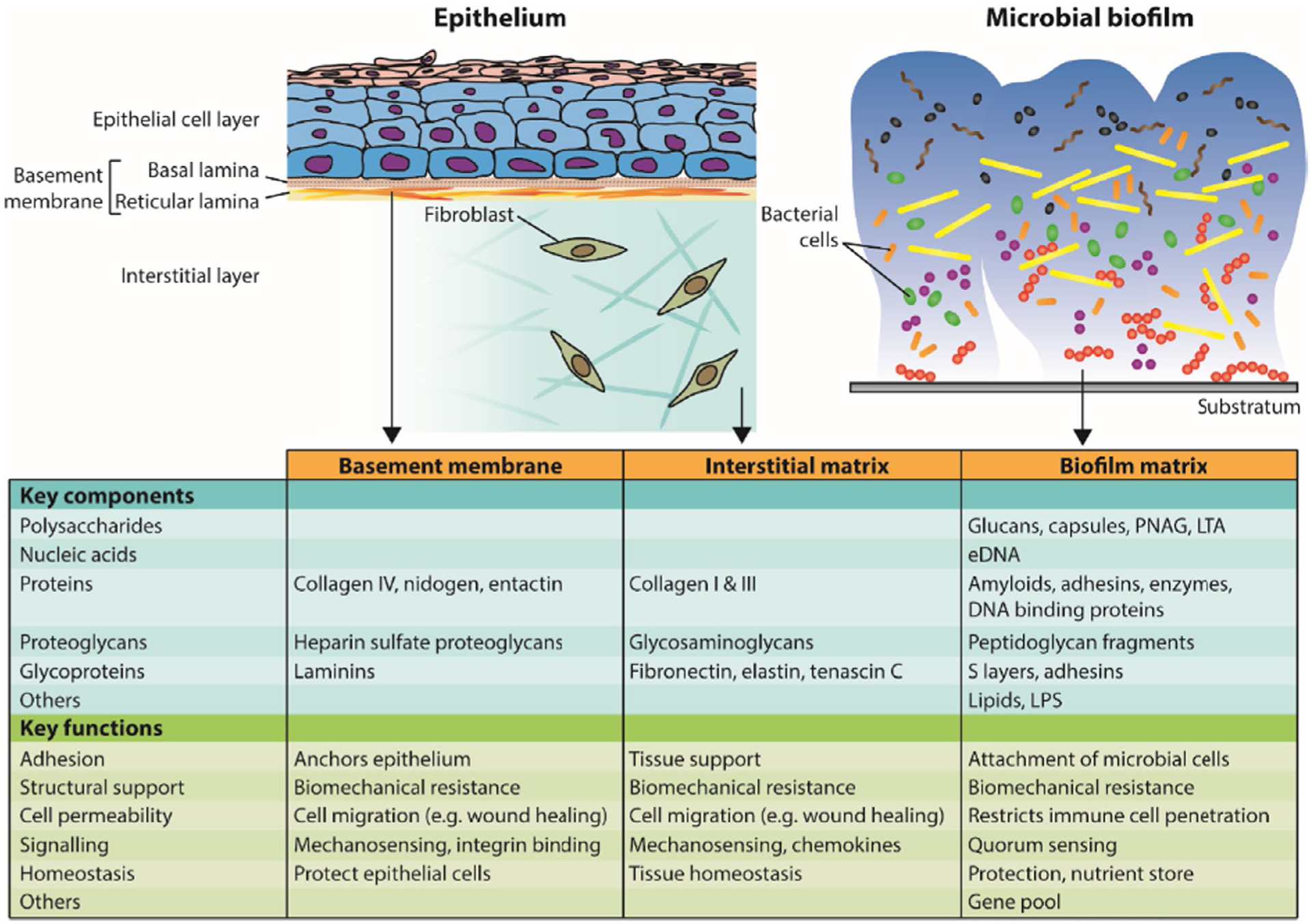
Comparison of the structure and function of host vs biofilm extracellular matrix. Host tissue contains a variety of different extracellular matrices. Shown here are the basement membrane and interstitial matrix underlying a layer of keratinized epithelium. Each of these contains proteins, proteoglycans, and glycoproteins that serve a variety of functions in adhesion, sensing, and protection. The basement membrane consists of the basal lamina, which forms the underlying layer for the epithelial cells, and the reticular lamina, composed primarily of collagenous fibers that serve to anchor the basal lamina. The interstitial matrix contains a variety of different cells, including fibroblasts. The biofilm matrix contains macromolecules primarily derived from the resident bacterial cells. These also function in adhesion, sensing, and protection, in addition to acting as a store of nutrient and a potential source of DNA for natural transformation. eDNA, extracellular DNA; LPS, lipopolysaccharide; LTA, lipoteichoic acid; PNAG, poly-*N*-acetylglutamic acid

Studies of biofilm matrices had been ongoing for many years before the term “biofilm” was first coined to describe interface-associated microbial communities in 1975.^1^ Early work on freshwater biofilms described the matrix as a “slime layer” and revealed polysaccharide-like material by electron microscopy.^2^ Intercellular material was also observed in dental plaque and was identified as polysaccharide based on reactions with osmium-black visualized under the electron microscope.^3^ The extracellular polysaccharide layer surrounding both prokaryotic and eukaryotic cells, and present in tissues as a basement membrane, was termed “glycocalyx” by Bennett in 1963.^4^ Glycocalyx means “sweet husk” and was considered appropriate since the layer always contained carbohydrate.^5^ In bacteria, two types of glycocalyx were identified: rigid paracrystalline S-layers surrounding bacterial cells, and more flexible capsules that may remain cell associated or be shed into the wider environment and form a casing for microcolonies.^6,7^ Thus, from an early stage, the same terminology was used to describe bacterial and eukaryotic extracellular matrices. The use of the term “glycocalyx” to describe the biofilm matrix has largely been dropped, since it overemphasizes the importance of carbohydrates. Nevertheless, it is clear that there are similarities in both structure and function between eukaryotic and bacterial extracellular matrices (Figure 1).

**FIGURE 2:**
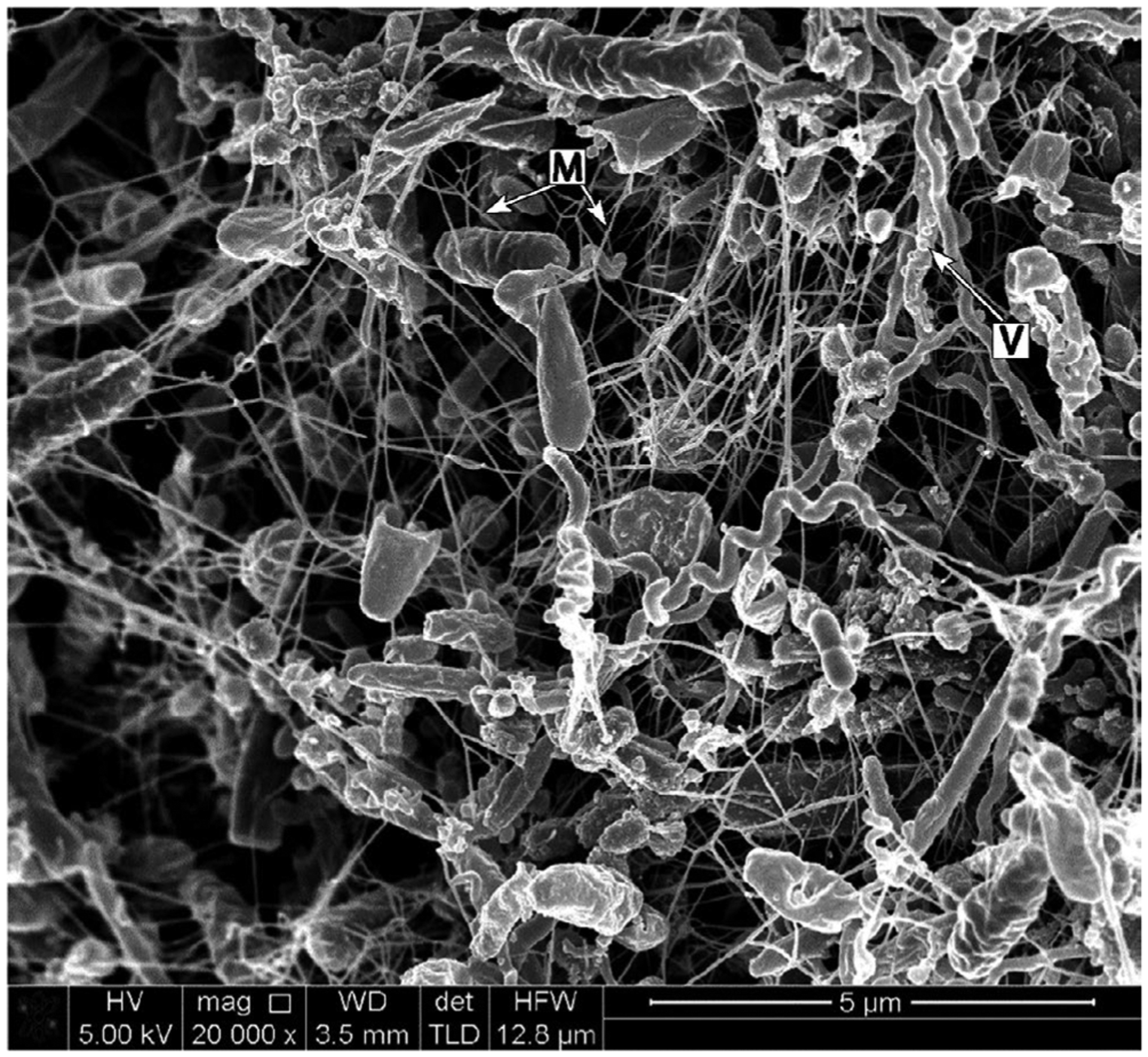
Scanning electron micrograph of subgingival dental plaque on the surface of a tooth extracted due to periodontal disease. Microbial cells are connected by a meshwork of fibrous material (M), apparently the collapsed remains of a hydrated polymeric matrix. Some microbial cells are associated with small particles or vesicles (V) that may provide a source for matrix polymers

Arguably the most thoroughly characterized biofilm matrix polymers in dental plaque are the glucan and fructan polysaccharides that are produced by the action of extracellular glucosyltransferase and fructosyltransferase enzymes on sucrose.^8^ These polysaccharides are considered to be important virulence factors in the pathogenesis of dental caries.^9,10^ There is also an extensive extracellular matrix in subgingival dental plaque of patients with periodontitis (Figure 2).^11,12^ The chemical composition of this matrix is not well understood at present. As with many microbial biofilms,^13^ the structural components of the matrix likely include a complex mixture of carbohydrates, proteins, nucleic acids, lipids, and other macromolecules derived from both the resident microorganisms in the biofilm and from the host. These polymers accumulate once the surface has become colonized with microbial cells. In the first 1–2 hours of colonization, bacterial cells are sparsely scattered over the enamel surface and there is little evidence of biofilm matrix.^14^ Small amounts of matrix material can be observed by electron microscopy from 2 hours onward.^15^ It is likely that the matrix undergoes changes during the transition from biofilms at the gum margins in health to subgingival dental plaque associated with periodontal disease. Identifying these changes and the key macromolecules that contribute to the function of pathogenic dental plaque may provide new targets for oral biofilm control. This article discusses the current state of knowledge regarding the dental plaque matrix. Where possible, we will focus on the microbial-derived matrix of subgingival dental plaque and its potential role in the pathogenesis of periodontal disease.

## 2 | KEY COMPONENTS OF THE MATRIX

The presence of a matrix is one of the defining features of microbial biofilms and is responsible for many of their emergent characteristics (the properties of the system that only appear when the cells are together in a biofilm).^16^ The major classes of extracellular polymeric substances that form the matrix are common to most biofilms and comprise carbohydrates, proteins, nucleic acids, and cell wall polymers, such as peptidoglycans and lipids.^13^ However, within these classes, there is extensive variation in the specific types and proportions of macromolecules between different types of biofilm. Analysis of the bulk chemical composition of the biofilm matrix requires large amounts of material, which is difficult or impossible to obtain for subgingival dental plaque. Therefore, much of our understanding of the biofilm matrix comes from in vitro studies on individual species or on targeted detection of specific macromolecules.^17^ Together, these methods are starting to reveal the complex macromolecular composition of the dental plaque extracellular matrix.

### 2.1 | Carbohydrates

**FIGURE 3:**
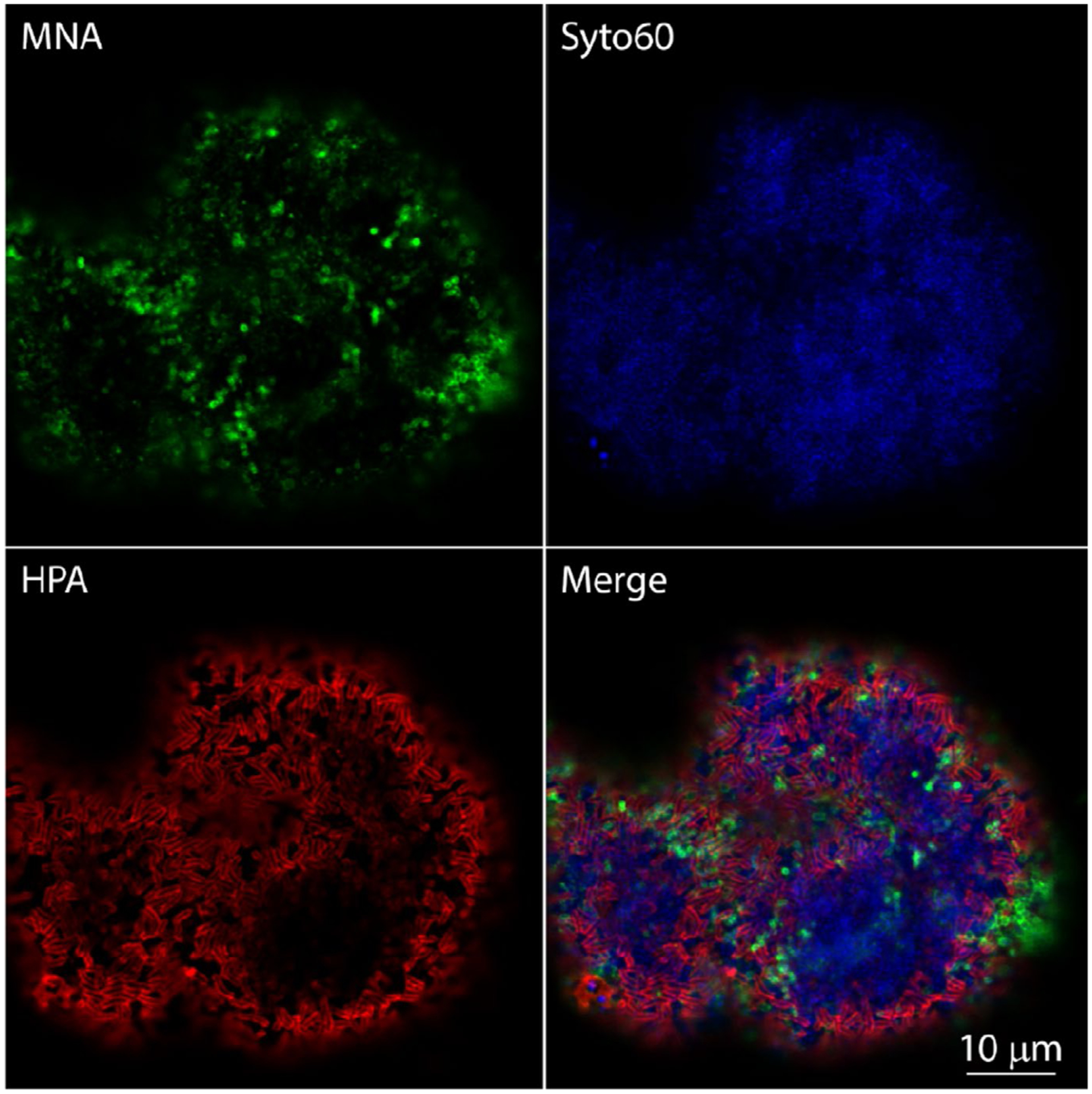
Fluorescent lectin binding analysis of supragingival dental biofilm, grown in situ in the mouth of a volunteer for 48 hours in the absence of dietary sucrose. The biofilm was stained with Morniga-G lectin (MNA-G-fluorescein isothiocyanate, green, recognizes galactose), *Helix pomatia* lectin (HPA-tetramethylrhodamine, red, recognizes *N*-acetyl-α-galactosamine), and Syto60 (blue, stains DNA). Maximum projection images are shown; scale bar: 10 μm. The target carbohydrates appear to be primarily associated with microbial cell walls. Image kindly provided by Thomas R. Neu, Pune N. Paqué, and Sebastian Schlafer

Carbohydrates constitute approximately 20% of the dry weight of supragingival dental plaque, and around two-thirds of these are water insoluble.^18,19^ A significant proportion of this biomass consists of intracellular storage polysaccharides and other intracellular carbohydrates.^20^ In addition, approximately 2%−10% of the dry weight of dental plaque consists of glucans, which are homopolymers of glucose that are produced extracellularly from sucrose by glucosyltransferase enzymes.^19^ Sucrose can also be converted to fructose polymers (fructans) by fructosyltransferases.^21^ Fructans tend to be present at lower amounts than glucans in dental plaque.^10^ Glucans and fructans can be observed macroscopically in sucrose-fed laboratory monocultures of oral streptococci as crystalline structures on surfaces of agar plates or suspended in planktonic cultures. When grown in the presence of sucrose, dental plaque has a significantly increased wet weight and increased concentration of alkali-soluble polysaccharide due to the production of glucans and fructans.^22,23^ The ubiquity of these polysaccharides, particularly in cariogenic oral biofilms, has led to them becoming the focus of intensive research for several decades, and they have been the subject of several comprehensive reviews.^8,21,24^ Water-insoluble glucans and fructans promote the attachment of bacteria to dental plaque, thus driving an increase in complexity of the population as plaque ages.^25–27^ However, subgingival dental plaque will become cut off from dietary nutrient sources, including sucrose, and therefore extracellularly synthesized glucans and fructans are not considered to play a major role in periodontal disease. Nevertheless, glucose is available in gingival crevicular fluid,^28^ and carbohydrates as a whole can be observed in dental plaque even in the absence of dietary sucrose. Using fluorescent lectin–binding analysis, a wide range of carbohydrate structures were detected in 48-hour supragingival dental biofilm grown in situ in the mouths of volunteers who abstained from ingesting sucrose.^29^ Some of these glycoconjugates appeared to be closely associated with the surfaces of microbial cells (Figure 3), whereas others were distributed more diffusely in the biofilm matrix.^29^

#### 2.1.1 | Protein-linked bacterial glycans: S-layers, outer membrane proteins and flagella

Though in its infancy compared with other areas of glycobiology, it is becoming increasingly obvious that attachment of glycans to surface proteins in bacteria is a common event rather than being a rare oddity.^30^ For example, since its initial discovery in Archaea, there are now examples of biologically important glycosylation events of bacterial surface proteins with import for biofilm formation that, given their surface location, should be considered part of the biofilm matrix. This is even more pertinent when considering our increasing awareness of bacterially derived vesicles that carry proteins and other surface molecules derived from the outermost membrane of either gram-positive or gram-negative bacteria.

As outlined later (Section 2.3), bacterial species contain a wide range of protein-based adhesins; these include well-characterized pili and fimbriae proteins, as well as bacterial flagella and amyloid-type proteins, such as the leucine-rich repeat family proteins of *Streptococcus* spp and *Actinomyces* spp, which are key species in oral biofilms.^31–33^ Indeed, it is now becoming clear that correct glycosylation is essential to the correct folding and function of these proteins.^34,35^ For example, loss of glycosylation of the Fap1 serine-rich repeat adhesin with GalNAc, GlcNAc, and rhamnose of the oral commensal *Streptococcus parasanguinis* influences biofilm-forming ability.^36–38^ Similarly, the leucine-rich–repeat adhesins of other oral primary colonizing organisms, such as *Actinomyces oris* GspA, lose biofilm adhesion capability if the glycosylation machinery that adds their glycan moiety is deleted.^39^ In addition to these extended-repeat–containing adhesins, there are also examples of shared glycosylation pathways between surface lipopolysaccharide glycosylation pathways and flagella proteins that are key in biofilms.^40^ These include well-studied flagellin glycosylation pathways, such as those in *Campylobacter* spp^41,42^ and *Aeromonas* spp.^34,43^ In fact, glycosylation of surface-biofilm–contributing proteins and structures in other gram-negative bacteria is also becoming increasingly understood, with numerous examples particularly evident for the keystone periodontal pathogen *Porphyromonas gingivalis*, such as the minor fimbriae (MfaI),^44^ the OmpA proteins,^45^ and the major gingipain virulence factors.^46^ However, this is by no means the only example in periodontitis virulence, with the EmaA collagen adhesin of *Aggregatibacter actinomycetemcomitans*^47^ key in biofilm adhesion, but also, one imagines, in adhesion involving the human collagen-rich extracellular matrix present on the hard and soft surfaces in the mouth.

Finally, bacterial proteinaceous surface-layers (S-layers) that exist across Archaea, gram-positive bacteria, and gram-negative bacteria are well known to be glycosylated.^48–50^ These are prominent in the biology of several oral bacteria, most notably for several *Campylobacter* spp^51^ and the periodontal pathogen *Tannerella forsythia*.^52^ Indeed, the S-layer of *T. forsythia* has been shown not only to be upregulated in biofilm cells,^53^ but also to be critical for adhesion to abiotic (plastic) surfaces^54^ and to cellular^55^ and mucin-coated surfaces.^56^ The interaction with mucins is strongly linked to its glycan modifications, with the host-mimicking nonulosonic sugars pseudaminic and legionaminic acid that terminate these glycans being key. Overall, it seems that the protein-based glycome of bacterial cells might be a critical part of the picture of surface interactions, with these glycans often defining hydrophilicity/hydrophobicity^57^ and charge characteristics of bacterial cells that may also be key for nucleation of other components, such as extracellular DNA (see later).^58^

#### 2.1.2 | Capsular and related polysaccharides

Many bacteria produce polysaccharide capsules that provide a variety of functions, including desiccation tolerance and protection from the immune system. Sometimes, the capsular polysaccharides remain tightly associated with bacterial cells. Alternatively, the polysaccharides may be released and become part of a more diffuse matrix. In either case, these polysaccharides are important extracellular components that contribute to the structure and function of biofilms. Genetic loci encoding capsular polysaccharide biosynthesis pathways similar to those of *Streptococcus pneumoniae* are present in the genomes of many oral streptococci, and immunodiffusion studies have shown that these are widely expressed.^59^ In general, the capsular polysaccharides form very thin layers on the surface of cells and are not easily visualized by microscopy. One exception is the *Streptococcus mitis* type strain (NCTC 12261), which produces a capsule that can be observed by electron or atomic force microscopy.^60^ Expression of the capsule reduces biofilm formation, cell-cell aggregation (autoaggregation), and epithelial cell binding, protects cells against phagocytosis and clearance in a mouse model of lung infection, and modulates sensitivity to host antimicrobial peptides.^60–62^ The thin layers of capsular polysaccharides present in *Streptococcus oralis*, *Streptococcus gordonii*, and *Streptococcus sanguinis* strains appear to be primarily involved in cell-cell adhesive interactions and have been termed “coaggregation receptor polysaccharides.”^63^ Many different streptococcal receptor polysaccharides have been characterized biochemically and shown to fall into different groups with distinct immunological and coaggregation specificity profiles.^63–65^ Species such as *A. oris* also produce polysaccharides involved in coaggregation, although the structure and synthesis of these are not well understood.^66^ In *Streptococcus mutans*, related polysaccharides known as rhamnose-glucose polymers are responsible for serotype specificity and biofilm formation.^67,68^ However, *S. mutans* undergoes very few coaggregation interactions, indicating that rhamnose-glucose polymers are not strong receptors for microbial cell-surface adhesins.

Capsules are important virulence factors for periodontal pathogens, such as *P. gingivalis*. At least six capsular (K) serotypes have been identified that vary in the extent to which they induce responses in dendritic cells and T-lymphocytes.^69,70^ In *P. gingivalis* W50, expression of the capsule reduced the activation of the host immune system, reduced phagocytosis, and increased virulence in a mouse abscess model compared with an unencapsulated mutant.^71^ Knocking out capsule production in *P. gingivalis* W83 led to enhanced autoaggregation and biofilm formation compared with the isogenic wild type.^72^ However, the presence of capsule is required for coaggregation with *Fusobacterium nucleatum* and for synergistic virulence in a mixed infection murine model of periodontitis.^73^ It is not yet clear whether capsules are strongly expressed within dental plaque. However, it is noteworthy that capsular biosynthesis genes in model commensal subgingival biofilms were upregulated in response to tobacco exposure.^74^ It is possible that the regulation of these extracellular carbohydrates may affect coaggregation interactions and drive the biofilm toward a more pathogenic state. Indeed, a DNABII protein regulates capsule expression in *P. gingivalis* intracellularly.^75^ This protein has an interesting dual role in the biofilm matrix, since it is also a structural component of the *P. gingivalis* extracellular matrix, as described later.

#### 2.1.3 | Poly-*N*-acetyl-d-glucosamine

Poly-*N*-acetyl-d-glucosamine was first identified as the polysaccharide intercellular adhesin that is an important component of the biofilm matrix of *Staphylococcus epidermidis*.^76^ More recently, poly-*N*-acetyl-d-glucosamine was identified as the primary adhesion-mediating polymer in biofilms formed by the periodontal pathogen *A. actinomycetemcomitans*.^77,78^ A proportion of poly-*N*-acetyl-d-glucosamine remains associated with the *A. actinomycetemcomitans* cell surface due to charge interactions with lipopolysaccharide. A de-*N*-acetylase, PgaB, is required for this interaction, and disruption of the catalytic domain of PgaB leads to reduced retention of poly-*N*-acetyl-d-glucosamine on the cell surface and decreased gene expression of the poly-*N*-acetyl-d-glucosamine biosynthesis gene locus.^79^ Further, this mutant did not form tenacious biofilms on the surface of glass tubes. Therefore, association of poly-*N*-acetyl-d-glucosamine with the cell surface appears to be important for attachment and colonization. Mutants that do not produce poly-*N*-acetyl-d-glucosamine show reduced virulence in a rat model of periodontitis, indicating that this polysaccharide is important for pathogenesis.^80^ In addition, the turnover of poly-*N*-acetyl-d-glucosamine modulates the positioning of *A. actinomycetemcomitans* in mixed-species biofilms. When co-cultured with *S. gordonii*, *A. actinomycetemcomitans* cells upregulated the *dspB* gene encoding the poly-*N*-acetyl-d-glucosamine–degrading enzyme Dispersin B.^81^ This resulted in cells becoming positioned at a distance from *S. gordonii* where they could benefit from metabolic cross-feeding by scavenging *S. gordonii–*derived lactate without succumbing to high concentrations of hydrogen peroxide in the close vicinity of *S. gordonii* cells. Therefore, production and turnover of the biofilm matrix appears to be an important factor in intermicrobial competition by *A. actinomycetemcomitans*.

#### 2.1.4 | Teichoic acids and lipoteichoic acids

Wall teichoic acids and lipoteichoic acids are highly charged glyco-polymers that are present in the cell walls of many gram-positive bacteria.^82^ Wall teichoic acids are covalently linked to peptidoglycan, whereas lipoteichoic acids are anchored in the cell membrane. The teichoic acid backbone consists of repeating units of negatively charged polyols, such as ribitol phosphate or glycerol phosphate, linked by phosphodiester bonds. In some cases, the repeating monomers are substituted with cationic d-alanyl esters, resulting in zwitterionic polymers. In *Staphylococcus aureus*, disruption of the *dltA* gene that is essential for d-alanine incorporation resulted in reduced colonization of inert surfaces, presumably due to the increased negative charge of the cell wall.^83^ In *S. epidermidis*, teichoic acids were identified in the extracellular fraction of cultures, and they were shown to be a critical component of the biofilm matrix.^84,85^ More recently, teichoic acids were identified as the major polysaccharides present in the matrix of *Listeria monocytogenes* biofilms.^86^

There is relatively little information at present on the role of extracellular teichoic acids or lipoteichoic acids in mixed-species dental plaque, even though lipoteichoic acids were shown to be abundant in sucrose-grown in vivo plaque as long ago as 1980.^87^ Studies on *S. mutans* demonstrated that lipoteichoic acids are present outside the cell and associated with glucosyltransferases.^88^ In fact, lipoteichoic acids inhibited glucosyltransferase activity, potentially leading to alteration in the glucan composition of biofilms. Lipoteichoic acids from *S. gordonii* were shown to associate with extracellular glucan polymers and to enhance the binding of *S. gordonii* cells to glucan aggregates.^89^ In addition, lipoteichoic acids from *Lactobacillus plantarum* inhibited the formation of *S. mutans* biofilms through suppression of exopolysaccharide production, and they reduced the formation of mixed-species oral biofilms in vitro.^90,91^ Interestingly, *S. mutans* proteins encoded by the *dltABCD* operon and responsible for d-alanylation of lipoteichoic acids were identified in biofilms by quantitative proteomics and were shown to be upregulated in three-species biofilms compared with monospecies biofilms.^92^ Lipoteichoic acids were detected in the matrix of both mixed-species and *S. mutans* monospecies biofilms.^93^ *S. mutans dltA* or *dltD* knockout mutants produced biofilms that were structurally distinct from those of the isogenic wild type and contained increased levels of lipoteichoic acids.^93^ These data indicate that lipoteichoic acids are important in the development of biofilms, particularly over the later stages during maturation of the matrix. It will be of interest to determine whether (lipo)teichoic acids are produced and secreted by gram-positive bacteria that are associated with the progression to periodontal disease, such as *Filifactor alocis* or *Peptoanaerobacter stomatis*.^94^ More generally, there is a need to characterize the extent and the origin of teichoic acids and lipoteichoic acids in the matrix of oral biofilms. It is not clear whether concentrations are sufficiently high to mediate intermicrobial competition, or whether certain species in the biofilm are particularly active in secreting these molecules. This information will provide a better understanding of the contribution of gram-positive bacteria to the structure and composition of dental plaque.

#### 2.1.5 | Fungal polysaccharides

Fungi are commonly present in the mouth, including in subgingival dental plaque.^95,96^ Of these, *Candida albicans* and other *Candida* spp have been most intensively studied, although molecular methods have detected many other genera.^97^ The biofilm matrix of *C. albicans* monocultures in RPMI medium consists of approximately 55% proteins, 25% carbohydrate, 15% lipids, and 5% nucleic acids.^98^ The polysaccharide component is largely composed of α-mannan, β−1,6 glucan, and β−1,3 glucan, which are major polysaccharides in the fungal cell wall.^99^ Interestingly, the glucans and mannan appear to be arranged in a complex that is assembled extracellularly.^100^ Similar extracellular mannan-glucan complexes are produced by a wide range of *Candida* spp, indicating that these are important components for *Candida* biofilm formation.^101^

### 2.2 | Extracellular DNA

Though the role of extracellular DNA in the extracellular matrix of oral biofilms is understudied, the work to date indicates that extracellular DNA acts as an architectural material in the maintenance of the structural integrity of biofilms. Indeed, there is evidence that at least the periodontal pathogens *A. actinomycetemcomitans*, *Prevotella* spp, and *P. gingivalis* can rely on extracellular DNA for the maintenance of their extracellular matrices.^12,102–104^

Extracellular DNA has been known to be part of the extracellular bacterial milieu for over 90 years, ever since Griffith^105^ discovered the *transforming principle* and Avery et al^106^ showed that extracellular DNA was horizontally transferred between *S. pneumoniae* strains. Later, several investigators discovered that extracellular DNA was a natural component of microbial mats (the ecologic precursor term for biofilms).^107–109^ However, it is only since 2002 that it has become clear that extracellular DNA is not only omnipresent in the extracellular matrix of bacterial biofilms but possesses a critical structural function as well. This was demonstrated when Whitchurch et al^110^ showed that deoxyribonuclease I could be used to prevent *Pseudomonas aeruginosa* biofilm formation. Human recombinant deoxyribonuclease I (dornase alpha) was already used as a mucolytic to alleviate the symptoms of cystic fibrosis,^111,112^ a condition that is often associated with chronic *P. aeruginosa* pulmonary infection. The observation that deoxyribonuclease can also control *P. aeruginosa* biofilms has opened the door to optimizing deoxyribonuclease as a therapeutic to control the microbial infection.^113^ Though the removal of biofilms by deoxyribonuclease enzymes has been recapitulated in multiple single and mixed-species biofilms, including dental plaque,^12,102,107,108,114–116^ the biofilms eventually become resistant to deoxyribonuclease treatment as they age. Initially, this was interpreted to mean that the DNA had turned over in favor of other matrix materials or that extracellular DNA was only required for initial biofilm formation. More recently, it has become clear that the extracellular DNA is consistently present in the biofilm matrix as the biofilm matures and that, where examined, it is even more critical to biofilm stability.^117^ Indeed, as the biofilm matures, the extracellular DNA enters a nuclease-recalcitrant state, likely to further protect the resident bacteria in the biofilm. Importantly, DNA has several relevant qualities that make it a versatile matrix material. First, it is omnipresent in eubacteria^118^ and microbial fungi,^98,119^ and is therefore accessible to all microorganisms whether in single or mixed-species biofilms. Second, it is relatively stable at varying pH extremes,^120^ like those that occur in the oral cavity. Third, it has a significant persistence length of ~50 nm, which means it is stiff over this short distance but can bend over longer distances.^121^ Fourth, since DNA can base pair between strands, it has the capacity to form complex secondary structures, including fibers.^122,123^ Further, as already mentioned, extracellular DNA becomes resistant to nuclease digestion as biofilms mature, thus protecting the resident bacteria from external hazards.

The presence of extracellular DNA has been documented in multiple single and mixed-species oral biofilms.^11,12,102,103,107–110,116,124–127^ Indeed, many oral streptococci rely on extracellular DNA for biofilm matrix structural stability,^12,116,127^ and in some cases extracellular DNA improves adherence to the tooth surface.^128,129^ In contrast, few periodontal pathogen biofilms have been thoroughly examined for their reliance on extracellular DNA. Of those that have been examined, *A. actinomycetemcomitans*, *Prevotella* spp, and *P. gingivalis* have been shown to rely on extracellular DNA for wild-type matrix formation.^102,114,116^ In addition, *Enterococcus faecalis*, which is associated with secondary endodontic infections, is also reliant on extracellular DNA.^124^ Other “red complex” pathogens, like *Treponema denticola* and *T. forsythia*, have yet to be examined.

The apparent reliance of biofilm matrix stability on extracellular DNA indicates that extracellular DNA is a universal matrix material. This makes sense, as it is one of the few structural commodities that is available to all microorganisms (ie, eubacteria and fungi). According to this hypothesis, when microorganisms need to enter or be inclusive in a multispecies biofilm, they could use extracellular DNA as the common structural material. This would also imply that the structure of the extracellular DNA would need to be sufficiently conserved. Indeed, images detailing the structure of extracellular DNA from multiple species are remarkably similar,^125,130^ showing a three-dimensional lattice of DNA. Though this could imply a random self-forming structure, it is more likely that this structure would need to be sufficiently robust so as to be stable under varied conditions. In this regard, other ubiquitous components would need to help shape and stabilize the extracellular DNA architecture. Indeed, it has recently been shown that the vertices of these scaffolds are functionally related to Holliday junction recombination intermediates, structures that are both created and turned over by known DNA repair pathways in the cell.^131^ It has yet to be determined how the extracellular structures form, but they are critical for the integrity of the matrix.

### 2.3 | Proteins

A wide range of proteins have been identified in the matrix of microbial biofilms. For example, studies on *Vibrio cholerae* and *P. aeruginosa* biofilms have identified secreted proteins, cell surface adhesins, and subunits of pili and flagella in the biofilm matrix (reviewed by Fong and Yildiz^132^). Proteins are estimated to contribute greater than 50% of the biomass of *C. albicans* biofilms, and include enzymes in metabolic pathways for carbohydrates, amino acids, and energy metabolism.^98^ Proteomic analysis of plaque-like biofilm grown in vivo identified a variety of bacterial stress-response proteins in the extracellular matrix.^133^ Sucrose-grown biofilms contained higher levels of certain carbohydrate metabolism proteins, including pyruvate kinase and components of a mannose-specific phosphotransferase system, whereas control (no sucrose) biofilms contained higher levels of calcium-binding proteins. Amyloid fibers are important structural components of biofilms.^134,135^ Several proteins produced by *S. mutans* can form amyloids including the antigen I/II adhesin (P1 or PAc), WapA, and SMU_63c.^136^ It is not yet clear how these proteins affect the structure of the dental plaque matrix, or which other amyloid proteins are present in the matrix.

Amyloid-forming proteins, such as antigen I/II, also play important roles in adhesion of streptococcal cells to bacterial or host receptors.^137^ A number of additional adhesins are produced by oral streptococci, including serine-rich repeat proteins, lipoproteins, and pili.^138^ These proteins are critical for adhesion and colonization, the first steps of biofilm formation. Type IV pili have recently been discovered in *S. sanguinis* and have been shown to mediate twitching motility in some strains.^139^ *S. sanguinis* SK36 produces short hair-like type IV pili that do not confer motility but are important for adhesion to host cells.^32,140^ Gram-negative oral bacteria also produce a variety of adhesins that contribute to mixed-species biofilm formation and host cell interactions. For example, long (FimA) and short (MfaI) pili of *P. gingivalis* are involved in coaggregation and adhesion to host cells.^141–143^ In addition, leucine-rich repeat proteins, such as BspA of *T. forsythia*, are important adhesins for host receptors, such as glycoprotein340, and contribute to bone loss in animal models of periodontitis.^31,144^ However, BspA is downregulated in biofilms, possibly in order to evade immune recognition.^145^

Extracellular enzymes are key components of biofilm matrices, including dental plaque. Enzymes that catalyze biosynthetic processes are rare due to the lack of an energy source outside the cytoplasmic environment. The major exceptions in oral biofilms are the glucosyltransferases and fructosyltransferases, described earlier, that harness the energy of the glycosidic linkage in sucrose to synthesize glucan or fructan polymers.^146^ Degradative enzymes, such as proteases, deoxyribonucleases, and glycosidases, are secreted by many bacteria and play a variety of different roles, including turnover of the matrix, scavenging of nutrients, and modulating host immune responses. For example, cysteine proteases of periodontal pathobionts (such as *P. gingivalis*, *T. denticola*, and *T. forsythia*) target host proteins (including complement components, cytokines, and matrix metalloproteinases), leading to the destruction of host tissues and the progression of periodontal disease.^147,148^ Bacterial proteases have also been implicated in processing cell-surface proteins and in bacterial cell-cell sensing.^147,149^ However, the role of microbial proteases in processing structural biofilm matrix proteins, including functional amyloids, is not yet clear. It is interesting to note that *P. gingivalis* proteases (gingipains) have been detected in association with amyloid plaques in the brain tissue of Alzheimer’s patients, and it is possible that they play a role in stimulating amyloid formation.^150^ Extracellular deoxyribonucleases are produced by many oral bacteria and may be important in biofilm matrix turnover, particularly in the early stages of biofilm formation when extracellular DNA is most sensitive to degradation.^104^ Extracellular glycosidases, such as Dispersin B and fructanases, target microbially synthesized polysaccharides,^151,152^ whereas enzymes such as neuraminidases (sialidases) primarily target host components.^153,154^ Several distinct glycosidase activities for nutrient scavenging have been identified in oral bacteria.^153,155–159^ A range of glycosidase activities from the pooled resources of several species in the oral biofilm, acting in concert with proteases, are required for the degradation of complex glycoproteins in saliva, which serve as a key nutrient source during the development of dental plaque.^160,161^

**FIGURE 4:**
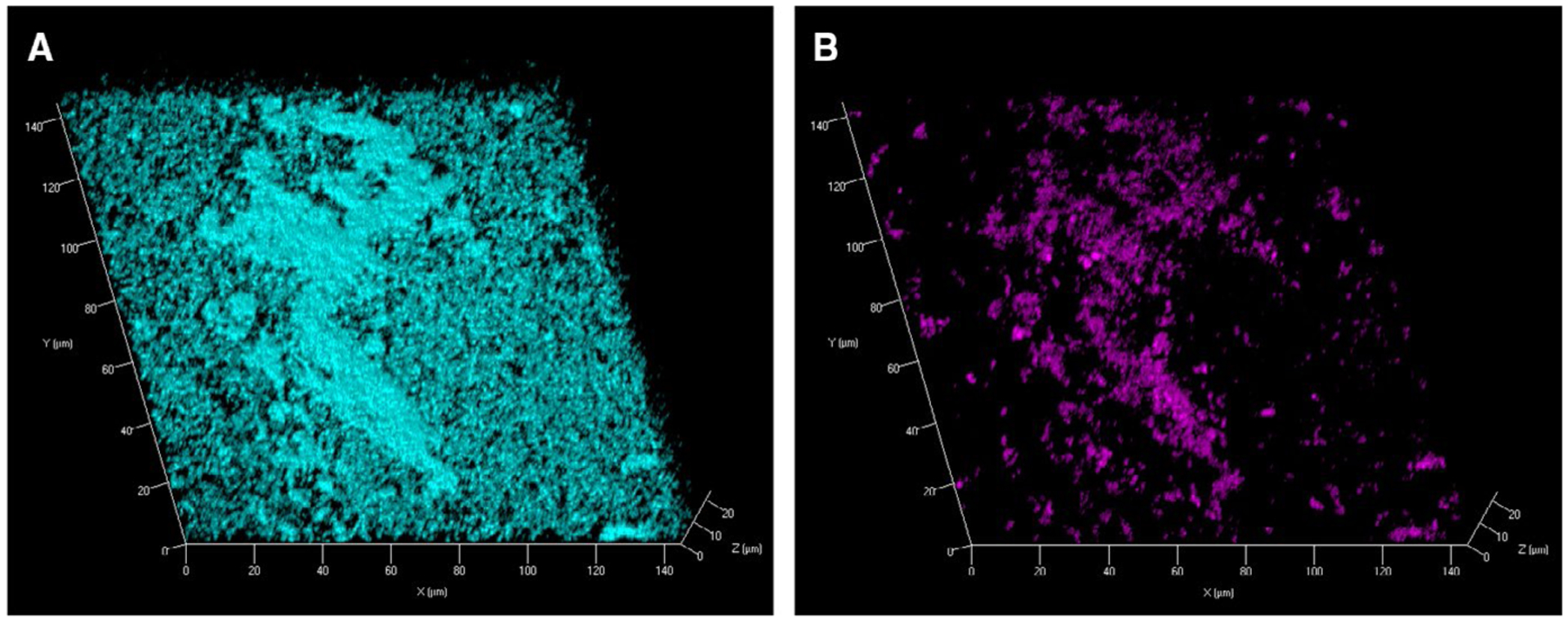
*P. gingivalis* histone-like protein PG0121 is abundant in dual-species biofilms with *Streptococcus gordonii* (*Sg*). *Porphyromonas gingivalis* (*Pg*) was seeded into extant *S. gordonii* biofilms and immunofluorescence was performed to determine the distribution of PG0121 present in dual-species biofilms of a 1:1 ratio of *Pg*:*Sg*. All bacterial cells were labeled with the membrane stain FM4-64 and pseudocolored cyan (A), and PG0121 was detected with antibodies directed against PG0121 followed by the addition of secondary antibodies conjugated to Alexa Fluor-488 and pseudocolored magenta (B)

Many proteins interact with other molecules in the biofilm matrix, such as extracellular DNA. Proteins that would manipulate the extracellular DNA structure fall into one of three categories: inducing structure, stabilizing structure, or altering structure. Proteins that could affect extracellular DNA structure would be those that naturally bind, induce, and/or maintain DNA structure. Eubacteria possess nucleoid-associated proteins that make up the intracellular chromatin. Though nucleoid-associated proteins vary between genera, one family of nucleoid-associated proteins is absolutely conserved: the DNABII proteins.^162^ All eubacteria possess at least one allele of the DNABII family.^162^ All DNABII proteins are homologous and function as homo or heterodimers. Importantly, the DNABII proteins bind with high affinity to DNA that is bent and, as a consequence, stabilize the DNA structure. Recently, it was shown that multiple single and mixed-species biofilms not only possess extracellular DNABII, but also that DNABII proteins are bound to the vertices of the lattice.^130^ Indeed, it was recently shown that these structures are functionally equivalent to Holliday junction recombination intermediates,^131^ where DNABII proteins are known to bind and stabilize these structures.^163^ Subsequent work showed that, in each case, biofilms could be disrupted and the resident bacteria released by titrating the DNABII protein from the matrix with an antibody directed against DNABII.^117,130,164–166^ Included in the biofilms tested were *A. actinomycetemcomitans*, *S. mutans*, and *P. gingivalis*, indicating that not only do potentially pathogenic oral bacteria possess extracellular DNA and DNABII proteins but they can also be targeted for biofilm resolution.^164,166^ One novelty is that all DNABII proteins examined to date are sufficiently antigenically similar such that an antibody directed to one will bind with high affinity to any other. The only exception so far is the DNABII protein PG0121 from *P. gingivalis*, encoding histone-like protein β-subunit, which is antigenically unique.^166^ Despite this distinctiveness, PG0121 can be complemented extracellularly by the DNABII protein from *S. gordonii*, showing that DNABII proteins appear to function in the biofilm matrix in an identical manner.^166^ This means that antibodies directed against PG0121 will only affect *P. gingivalis*. Indeed, PG0121 is abundant in dual-species biofilms with *S. gordonii* (Figure 4), and anti-PG0121 effectively prevents *P. gingivalis* from entering extant biofilms.^165^ This further proves that the nucleoprotein complex formed by extracellular DNA and DNABII proteins creates a common inclusive structure recognizable by bacteria.

Though it is formally possible that other nucleoid-associated proteins play a role in inducing or stabilizing, or perhaps simply altering, the extracellular DNA–dependent matrix (nucleoid), few candidates have emerged so far. Indeed, for nontypeable *Haemophilus influenzae*, a survey of extracellular nucleoid-associated proteins showed that, even when present, only the DNABII proteins affected the structural integrity of extant biofilms.^167^ There are exceptions, however. For example, beta toxin is released from *S. aureus* and binds to extracellular DNA. Though not a nucleoid-associated protein, it does facilitate formation of extracellular DNA fibers by cross-linking DNA and enhancing biofilm formation.^126^ A second exception is curli fibers from *Escherichia coli* and *Salmonella*.^168^ These proteins are synthesized extracellularly to create an amyloid that binds to extracellular DNA that facilitates biofilm formation. Examples of similar proteins from oral bacteria have yet to be discovered, but the aforementioned examples posit the possibility that each bacterium may have a means to manipulate the extracellular DNA nucleoid. Finally, though proteins that induce and maintain extracellular DNA–dependent matrix structure beyond the DNABII protein are rare, nucleases that cleave DNA are not. Indeed, many bacteria release nucleases, including oral pathogens such as *Prevotella* spp, *S. mutans*, *P. gingivalis*, *T. forsythia* and *F. nucleatum*.^104,169,170^ Interestingly, the role of these nucleases has been ascribed not to altering the bacteria’s own extracellular DNA but to disrupting neutrophil extracellular traps.^171^ This suggests that the extracellular DNA lattice possessed by bacterial biofilms differs sufficiently from that of neutrophil extracellular traps that only the neutrophil extracellular trap DNA is susceptible. Indeed, DNABII proteins are only found in eubacteria and, as such, appear to facilitate a recalcitrant extracellular DNA structure of just biofilm extracellular DNA. This tête-à-tête at the interface between biofilm and neutrophil extracellular trap DNA looms as the front line in host-pathogen interactions.

### 2.4 | Cell wall fragments

The outer layers of bacteria contain macromolecules, such as peptidoglycan, phospholipids, and, in the case of gram-negative bacteria, lipopolysaccharides, that may integrate into the biofilm matrix following cell lysis or the production of vesicles (see later). It is relatively difficult to study these molecules in the extracellular milieu; consequently, their role in biofilms is not well characterized. Wall-less (L-form) *Enterococcus faecium* are capable of attaching to solid substrata, indicating that peptidoglycan is not essential for the initial attachment phase of biofilm formation.^172^ However, peptidoglycan fragments may play roles in more mature biofilms as environmental cues, since it has been demonstrated that they regulate processes such as the germination of spores in *Bacillus*, the production of antimicrobial compounds by *P. aeruginosa*, and the yeast-hyphal transition in *C. albicans*.^173–175^ In addition, peptidoglycan is sensed by host cells through toll-like receptor 2 and nucleotide-binding oligomerization domain–like receptors and acts synergistically with lipopolysaccharides to induce bone resorption and osteoclastogenesis in mouse models of periodontitis.^176,177^ Lipids and lipopolysaccharides will likely aggregate into vesicles or associate with cell membranes in the hydrophilic environment of the biofilm matrix. Vesicles have been observed in dental plaque, and their role in delivery of macromolecules to the biofilm matrix is discussed later.^178^ The polysaccharide O-antigen of lipopolysaccharide has been shown to inhibit biofilm formation by a range of enteric gram-negative bacteria, and it is possible that this component may influence periodontal biofilms that are typically enriched in gram-negative species.^179^

### 2.5 | Host molecules

Although this article focuses on microbial-derived biofilm matrix molecules, it is important to note that natural dental plaque will also contain macromolecules of host origin. Components of saliva, such as proteins and glycoproteins, are continuously adsorbed onto surfaces. On the surface of enamel, these form the acquired enamel pellicle.^180^ More recently, it has been shown that there is also an extensive mucosal pellicle on the surface of oral soft tissues.^181^ Many of the (glyco)proteins that adsorb to oral surfaces also interact with oral microorganisms, including glycoprotein340, secretory immunoglobulin A, mucins, proline-rich proteins, amylase, and statherin.^182,183^ When dental plaque grows below the gumline, it becomes isolated from saliva and, instead, is exposed to gingival crevicular fluid, a serum exudate. This is rich in protein and bound glycans that can be released and bound by bacteria and is likely a source of molecules in the matrix of subgingival dental plaque.^184^ Periodontal pockets contain a variety of host inflammatory cells that may provide an additional source of proteins, glycoproteins, nucleic acids, and lipids for dental plaque.

### 2.6 | Interactions between macromolecules

**FIGURE 5:**
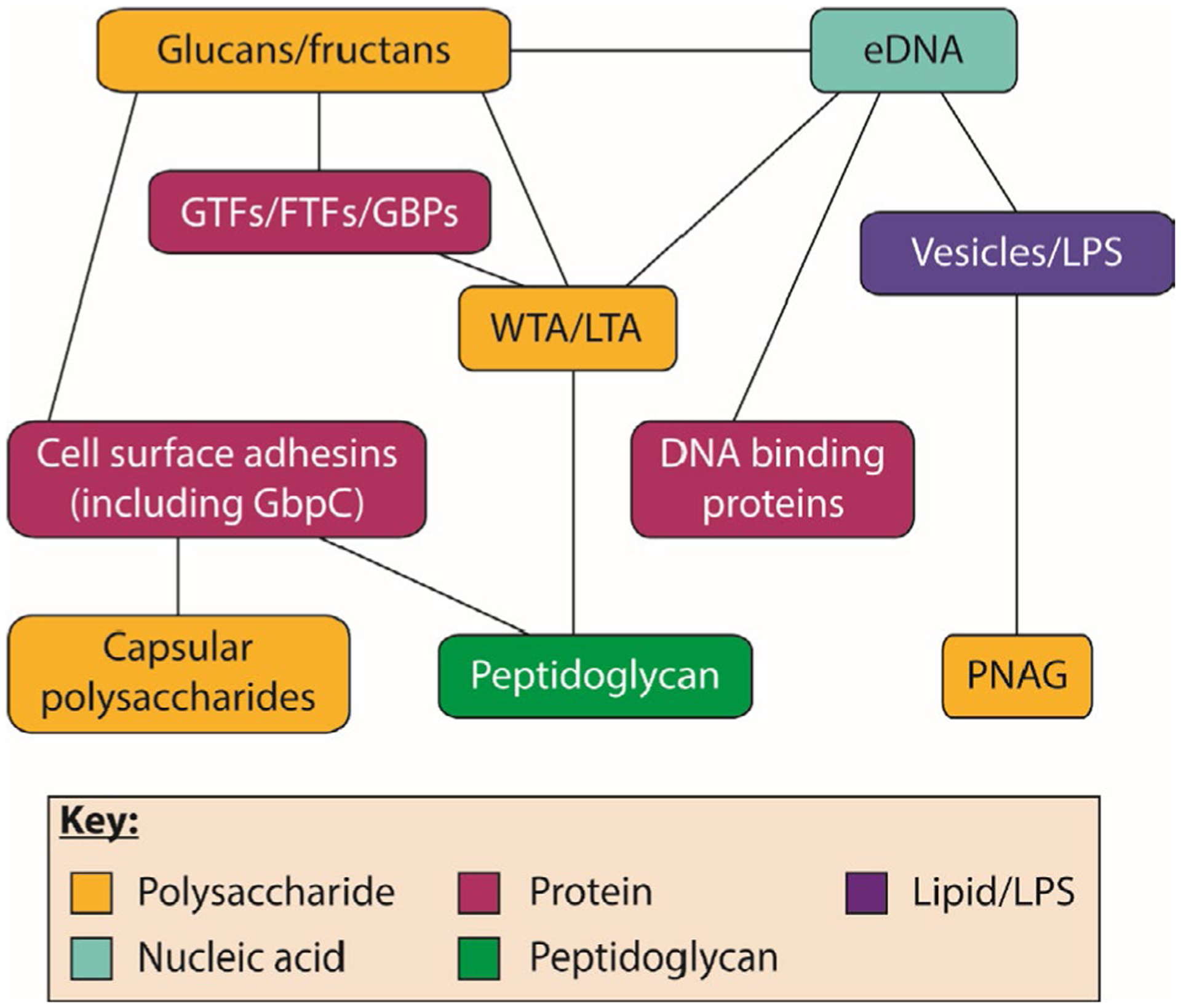
Interactions between macromolecules in the matrix of dental plaque. Glucans and fructans are associated with the enzymes that produce them (glucosyltransferases, GTFs/fructosyltransferases, FTFs), and insoluble glucans are recognized by glucan-binding proteins (GBPs), including the wall-anchored protein GbpC. Glucans and GTFs associate with extracellular DNA (eDNA) and lipoteichoic acid (LTA), which also binds extracellular DNA. In addition, wall teichoic acids (WTAs) and certain gram-positive cell-surface proteins are covalently linked to peptidoglycan. Therefore, these will remain attached to peptidoglycan fragments in the biofilm matrix. Cell-surface adhesins mediate coaggregation through binding to capsular polysaccharides. Proteins are also associated with extracellular DNA, and extracellular DNA is present on the surface of gram-positive membrane vesicles. Gram-negative outer membrane vesicles contain lipopolysaccharides (LPS), which bind to poly-*N*-acetyl-d-glucosamine (PNAG) through charge interactions

Throughout this review, multiple matrix molecules are described and discussed. Many of these macromolecules interact covalently or noncovalently to form complexes (Figure 5). For example, peptidoglycan of gram-positive bacteria is covalently linked to wall teichoic acids and to a variety of cell-surface proteins that contain conserved C-terminal motifs that are recognized and processed by sortase enzymes.^185,186^ Peptidoglycan fragments in the biofilm matrix will likely retain these interactions. Certain cell-surface adhesins, such as antigen I/II, WapA, and GbpC, form amyloids. It is not clear whether these amyloids remain bound to fragments of peptidoglycan in the matrix. Electrostatic interactions are responsible for many of the complexes that are formed, including between teichoic acids and proteins/polysaccharides/extracellular DNA and between poly-*N*-acetyl-d-glucosamine and lipopolysaccharides. Extracellular DNA is a key component of many biofilms and undergoes a number of interactions with other macromolecules. However, as already described, though periodontal pathogens make exopolysaccharides, for most of them it is unclear at present if they interact with or are exclusive of extracellular DNA. When grown in the presence of sucrose, the large quantities of exopolysaccharides produced by streptococci rival extracellular DNA in proportion and could, in principle, dominate, add to, or synergize with the extracellular nucleoid. For *S. mutans*, it is clear that insoluble glucan interacts productively with extracellular DNA to contribute to the extracellular matrix.^127^ Lipoteichoic acids are a large component of the gram-positive cell wall, and at least for *S. mutans* appear to interact with extracellular DNA in concert with glucans.^127^ More generally, extracellular DNA has been shown to associate with the surface of membrane vesicles.^187^ It is possible that other components of the outer layers of microbial cells, including lipids and lipopolysaccharides, may bind to extracellular DNA or other biofilm matrix components and alter the function of the matrix. As the field of the extracellular nucleoid evolves and mixed-species biofilms are analyzed, other matrix molecules that are exclusive to single species may not only be found to interact with extracellular DNA but, in the context of the mixed-species community, may be shown to interact with the extracellular DNA–dependent matrix at large.

## 3 | ORIGINS OF THE MATRIX

**FIGURE 6:**
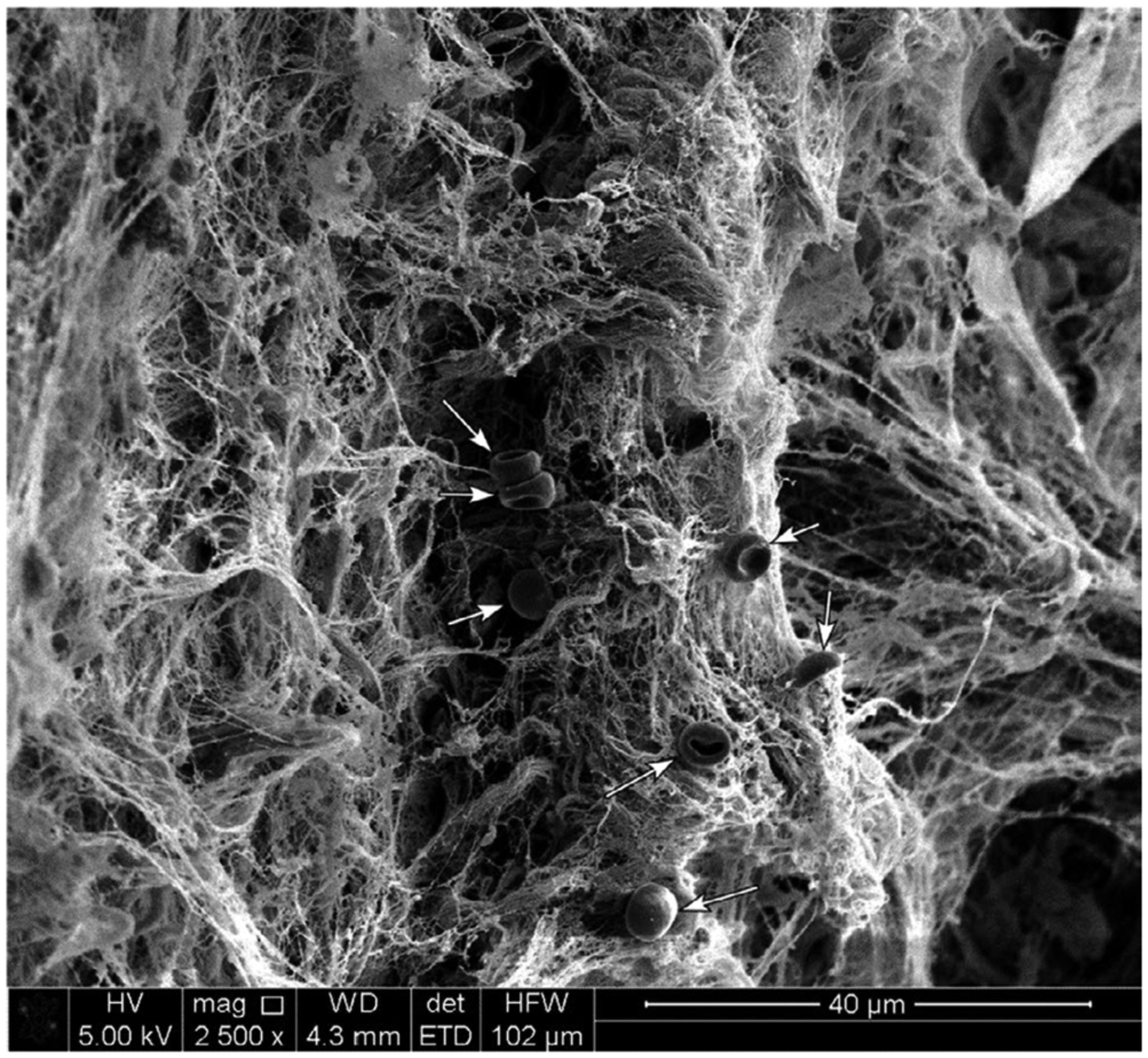
Scanning electron micrograph showing erythrocytes and fibrous material on the surface of a tooth extracted due to periodontal disease. Erythrocytes (arrows) are associated with fibrous noncellular material overlying the dental plaque on the tooth surface. Bacterial cells are not visible in this image

Many components of the dental plaque extracellular matrix are originally synthesized within microbial cells and actively secreted into the surrounding milieu. Microbial vesicles and cell lysis also play important roles in the accumulation of matrix material. Bacteriophages are abundant within dental plaque, and virus-like particles can be observed in association with microbial cells by transmission electron microscopy.^188,189^ These may provide a source of extracellular nucleic acids or proteins. In addition, molecules from the host diet, such as fiber, polysaccharides, and proteins, may become entrapped within dental plaque. These components, along with debris from nondietary sources, can be identified in dental calculus from archaeological specimens.^190^ Saliva is particularly important during the early phases of dental plaque growth, since molecules from saliva adsorb onto tooth surfaces and promote microbial attachment and biofilm formation.^191^ Depending on the location of dental plaque (above or below the gumline), macromolecules from saliva and/or gingival crevicular fluid may attach to dental plaque and integrate into the biofilm.^180^ In addition, host cells are commonly found in dental plaque and may contribute to the plaque matrix when they degrade. Epithelial cells from the tongue, oral mucosa, or gingiva can be found in dental plaque within 1 hour after introducing a clean tooth surface into the mouth.^192^ Periodontal disease and gingival bleeding lead to the accumulation of erythrocytes at or close to sites of dental plaque (Figure 6). For some immune cells, lysis of cellular contents appears to be an important process to generate extracellular “traps” containing nucleic acids, antimicrobial peptides, and proteins that catch invading microorganisms. The process of extracellular trap formation was first identified in neutrophils, and this has been shown more recently in macrophages.^171,193^ Neutrophil extracellular traps have been identified in purulent exudate from periodontal pockets of patients with chronic periodontitis.^194^ Proteins associated with neutrophil extracellular traps have also been identified in supragingival plaque during an experimental gingivitis study.^195^ The impact of nonmicrobial components of dental plaque matrices may be missed when dental plaque is modelled and studied in vitro. Future studies are needed to elucidate the complex network of microbial and nonmicrobial products in dental plaque in vivo.

### 3.1 | Secretion

Polysaccharides and proteins are actively secreted from microbial cells through a number of different export pathways. In some cases, DNA may also be exported through the type IV secretion system.^196^ For example, the *Neisseria gonorrhoeae* type IV secretion system exports single-stranded DNA into the surrounding milieu and is required for biofilm formation.^197^ Similarly, nontypeable *Haemophilus influenzae* has been shown to release DNA through a competence-mediated T4SS-like complex. In this case, the export of DNA was shown to contribute directly to the DNA-based extracellular polymeric material.^198^ However, it is not yet clear whether type IV secretion systems, or even whether single-stranded extracellular DNA, are important for oral biofilms. A brief overview of carbohydrate and protein export pathways will be presented here. For further information, the reader is referred to excellent reviews on the secretion of polysaccharides^199,200^ or proteins.^201–203^

Three major pathways are responsible for the export of polysaccharides in both gram-positive and gram-negative bacteria: (a) Wzx/Wzy dependent, (b) adenosine triphosphate–binding cassette dependent, and (c) synthase dependent. The Wzx/Wzy-dependent pathway mediates the export of lipopolysaccharide O-antigen polysaccharides and capsular polysaccharides.^204,205^ Central to this pathway are the Wzx flippase and the Wzy polymerase that elongate the polysaccharide chain on the outer surface of the cell. The polysaccharide is delivered to Wzx attached to the lipid acceptor moiety undecaprenyl phosphate.^206^ By contrast, adenosine triphosphate–binding cassette transporter systems translocate the fully formed polysaccharide using energy from ATP hydrolysis. This pathway is responsible for export of *S. mutans* rhamnose-glucose polymers.^207^ The synthase-dependent pathway involves the simultaneous polymerization and translocation via a membrane-embedded glycosyl transferase, and is employed for the export of poly-*N*-acetyl-d-glucosamine from bacterial cells.^200^ A similar pathway mediates the export of glucans in *C. albicans*.^208^

As in other eukaryotes, protein secretion in *C. albicans* is driven by vesicle-mediated trafficking between cellular compartments, and out to the cell surface.^209^ However, the secretory apparatus differs between yeast and hyphal cells, indicating that different morphologic forms may play distinct contributions to the extracellular proteome. Numerous protein secretion systems have been described in bacteria, and there is a great deal of confusion regarding their nomenclature in the literature.^202^ Bacteria with a gram-negative structure must transport proteins across two membranes to reach the extracellular milieu, whereas gram-positive bacteria only have to secrete proteins through the cytoplasmic membrane. Therefore, the Sec system, which is present in both gram-negative and gram-positive bacteria and transports proteins across the cytoplasmic membrane, is only a true secretion system in gram-positive organisms. In gram-negative bacteria, additional secretion apparatus is required to translocate proteins across the periplasmic membrane. The Sec and Tat systems are dependent on signal peptides that are present on the N-terminus of proteins.^210^ The key difference between these pathways is that Sec translocates unfolded proteins whereas Tat recognizes folded proteins. Signal peptides that are cleaved by the prepilin-specific signal peptidase PilD direct the transport of type IV pilus subunits in certain gram-positive bacteria, including *S. pneumoniae*.^211^ In gram-negative bacteria, nine different secretion systems have been identified that mediate export across the periplasmic membrane (type I-IX secretion system, designated T1SS-T9SS).^202,203^ Of these, T1SS, T3SS, T4SS, and T6SS span both membranes and transport proteins directly from the cytosol to the extracellular environment. The other systems traverse the periplasmic membrane only and require the prior export of proteins to the periplasm by Sec, Tat, or holins. T9SS was only discovered relatively recently and has been characterized in particular in periodontal pathobionts such as *P. gingivalis*, where it is responsible for secretion of a range of virulence factors including cysteine proteases,^203^ and *T. forsythia*, which employs T9SS for the secretion and assembly of S-layers.^52,212^

### 3.2 | Vesicles

Extracellular vesicles are abundant in dental plaque (Figure 2) and are produced by many species of bacteria and fungi. There are marked differences between vesicles from gram-negative and gram-positive bacteria. Gram-negative outer membrane vesicles, including those of *P. gingivalis*, contain lipopolysaccharides and protect DNA within them.^213,214^ By contrast, gram-positive vesicles, such as those from *S. mutans*, are associated with extracellular DNA on the external surface.^215^ The fungus *C. albicans* also produces extracellular vesicles that play a central role in biofilm matrix production by transporting proteins and polysaccharides (glucans and mannans) out of the cell.^216^ Approximately 45% of the proteins identified in the *C. albicans* biofilm matrix were also present in the proteome of biofilm extracellular vesicles, indicating that extracellular vesicles may be a major route for secretion of proteins into the matrix. Proteomics has also been extensively employed to characterize bacterial extracellular vesicles, and the proteins identified have been cataloged in a database (http://evpedia.info).^217,218^ In the gram-negative model biofilm organism *P. aeruginosa*, the extracellular vesicle proteome contained approximately 20% of the proteins found in the biofilm matrix, again indicating that extracellular vesicles may play a key role in delivering proteins to the matrix.^219^

Proteomics has been employed to analyze the proteins present in extracellular vesicles from several gram-negative periodontal pathobionts. For example, extracellular vesicles of *A. actinomycetemcomitans* are enriched in virulence factors, including the leukotoxin LtxA and the tight adhesion proteins TadA, TadD, TadE, TadF, TadG, and TadZ.^220,221^ Virulence factors are also abundant in *P. gingivalis* extracellular vesicles, which are enriched in T9SS substrates, including arginine and lysine-specific gingipains.^222^ *T. forsythia* extracellular vesicles are also enriched in substrates of T9SS, including S-layer proteins TfsA and TfsB.^212^ Interestingly, vesicles of *A. actinomycetemcomitans*, *P. gingivalis*, and *T. denticola* also contain a range of small ribonucleic acids that can be delivered directly to host cells and may play roles in immunomodulation.^223^

### 3.3 | Cell lysis

As with extracellular vesicles, electron microscopy studies have provided evidence of cell lysis within dental plaque.^11,12^ Lysis may occur as a natural consequence of cell senescence or may be induced by molecules that degrade the cell wall—including exogenous compounds, such as antibiotics or bacteriocins—or enzymes (autolysins) present within the bacterial cell. It appears that cell lysis is an important mechanism for delivery of extracellular DNA into the biofilm matrix. For example, competence-stimulating peptide–mediated induction of bacteriocins in *S. mutans* leads to lysis of a proportion of cells in a population and enhancement of the biofilm matrix by extracellular DNA.^224^ Competence-inducing peptide also triggers cell lysis in *S. mutans*, which will release DNA.^225^ The autolysin AtlA is critical for *S. mutans* extracellular DNA release and biofilm matrix production in vivo in a rat model of endocarditis.^226^ Fratricide is also responsible for extracellular DNA production and biofilm development by *E. faecalis*, an important endodontic pathogen.^227,228^ However, cell lysis is not essential for extracellular DNA in this species, as an abundant extracellular DNA matrix can be detected in early biofilms, where cell lysis is not detectable.^124^ In *P. aeruginosa*, explosive cell lysis of a subpopulation of cells, driven by the bacteriophage-like endolysin Lys, rapidly releases extracellular vesicles and liberates extracellular DNA into the biofilm matrix.^229^ It remains to be determined whether a similar process occurs in bacteria within dental plaque.

## 4 | FUNCTIONS OF THE MATRIX

The biofilm matrix is important for adhesion to substrata and for maintaining a homeostatic environment for the resident microbial cells.^134^ The matrix helps to position cells at a distance where mutually beneficial interactions are optimized and competition is minimized.^81,230^ Although the development of the biofilm matrix is considered to be a process that follows the initial colonization of an interface, there is evidence that matrix macromolecules are important for initial attachment of microorganisms. For example, cell-surface protein adhesins that are found within biofilm matrices are important for attachment to receptors in the saliva pellicle or for coaggregation interactions between microbial cells.^183^ In addition, extracellular DNA has been shown to promote the attachment of *S. mutans* to surfaces.^231^ In more mature biofilms, the matrix provides physicochemical forces for adhesion of the biofilm to the substratum and cohesion of the biofilm biomass.

### 4.1 | Adhesion/cohesion and mechanical resistance

The biofilm matrix acts as a focus for interactions between macromolecules (Figure 5), which help to retain cells within the biofilm and to stabilize the overall structure. For example, glucan binding proteins of *S. mutans* promote adherence to matrix glucans and shape the overall architecture of the biofilm.^25,26,232,233^ Biofilms are typical examples of multicomponent materials and exhibit viscoelastic behavior when subjected to external stress factors.^234^ Viscoelasticity consists of an elastic component that does not lose energy when a stress is applied and then removed, and a viscous component that undergoes molecular rearrangement in response to a stress and dissipates energy in the process. This means that the biofilm will deform under a given stress (eg, shear stress) but will return to a state that is similar to, but not necessarily identical to, the initial state after this given stress is removed.^234^ The mechanical properties of a biofilm and its sturdiness against detachment forces are further influenced by the shear forces that the biofilm experiences during growth.^13,234,235^ Biofilms grown under higher shear stress exhibit stronger attachment and stronger cohesive forces than biofilms grown under lower shear.^236,237^ This may be due to structural changes in the matrix; for example, with regard to the physical arrangement and structure of extracellular polymers, or due to a selection favoring subpopulations that produce biofilms with increased strength of their structural matrix under high-shear conditions.^13,238–240^ Although some of these studies were performed on biofilms from environmental pathogens, such as *P. aeruginosa*, the results may be translated to periodontal biofilms that grow under rather constant mechanical challenge exerted through the flow of naturally occurring fluids, like saliva or gingival crevicular fluid, or due to tongue movements.^234^ Furthermore, Paramonova et al^236^ showed that biofilms of typical oral colonizers *S. oralis* and *Actinomyces naeslundii* adapted to changes in hydrodynamic conditions by changing their architecture.

### 4.2 | Regulation of mass transfer and cell migration

The macromolecular biofilm matrix forms a scaffold that can impede the transfer of molecules and cells. Thus, poly-*N*-acetyl-d-glucosamine has been shown to restrict fluid convection and to retard the penetration of the quaternary ammonium compound cetylpyridinium chloride.^241^ Thurnheer et al^242^ found that penetration depths of macromolecules decreased linearly for molecular weights up to 240,000 Da in their Zurich biofilm model of supragingival plaque. The authors suggested that the discrepancy found between diffusion of these macromolecules in a biofilm compared with diffusion in bulk water most likely may be explained by the phenomenon of tortuosity. This means that a given molecule will be delayed in diffusion throughout a biofilm because its pathway is determined by the interstitial voids in the biofilm structure; consequently, the route will be a three-dimensional one rather than the direct path found for free diffusion in bulk water.^242^ It was also shown that the penetration of poly(ethylene glycol) with molecular weight of 10,000 Da through *S. mutans* biofilms was dependent on the density of the polymeric matrix of the biofilms, which was in turn influenced by the sucrose concentration in the culture medium.^243^ These authors concluded that steric exclusion is likely responsible for the decreased penetration. Although this concept seems quite intuitive, times needed for penetration throughout biofilms do not always increase with increasing molecular weight of the respective agents, even for chemically inert compounds. In fact, some studies have reported that even large antibiotics are able to penetrate in vitro biofilms within a few minutes.^244^ For instance, Oubekka et al,^245^ using time-lapse microscopy and fluorescence imaging, showed that BODIPY-labeled vancomycin penetrated to the deepest layers (~30 μm) of *S. aureus* biofilms in 8 minutes or less. On the other hand, Jefferson et al^246^ observed that penetration of BODIPY-labeled vancomycin through their *S. aureus* biofilms occurred rather slowly over the course of 60 minutes, which, however, may be related to the particular density of the extracellular polymeric substances in these biofilms due to overproduction of poly-*N*-acetyl-d-glucosamine by the respective *S. aureus* strain. This example demonstrates the difficulties with interpretation of in vitro studies on penetration of antimicrobials and other chemicals. In addition, the situation becomes more complex for charged molecules that undergo electrostatic interactions with biofilm matrix components.^247^ In this case, the biofilm matrix acts as an ion-exchange resin to reduce the rate of movement through the biofilm.^248^ For some molecules, such as strongly oxidizing agents, reaction with the outer layers of the biofilm can impede diffusion to the center of the structure. Therefore, models of mass transfer through biofilms need to account for both reaction and diffusion.^249^

Subgingival dental plaque is bathed in gingival crevicular fluid and is in contact with the gingival epithelium. Therefore, it is exposed to host immune mediators, including antibodies, complement, and immune cells. Mixed-species biofilms cultured in vitro retard the penetration of immunoglobulin G (IgG) and immunoglobulin M antibodies, to the point where IgG does not reach the center of clusters a few hundred micrometers in diameter.^242,250^ However, the diffusion of *S. mutans*–specific IgG was not affected by the presence of exopolysaccharides in single-species biofilms.^251^ It is possible that more complex matrix components or tighter cell-cell interactions in mixed-species biofilms are responsible for reduced levels of IgG penetration in these systems. Proteases present in the biofilm matrix may cleave antibodies, acting as “shared goods” to protect multiple species of bacteria within the biofilm. Immunoglobulin A–specific proteases are well known in oral streptococci, such as *S. mitis* and *S. sanguinis*,^252,253^ and *P. gingivalis* gingipains have been shown to cleave IgG.^254^ Similarly, proteases of periodontal pathogens cleave multiple complement proteins (reviewed by Damgaard et al^255^), which will potentially protect biofilm bacteria from host immunity.

Cell migration through host tissue extracellular matrices is critical for a wide range of physiologic processes and for responses to tissue damage (Figure 1).^256^ In a similar manner, bacteria can potentially migrate within biofilms. Motility mediated by type IV pili and flagella is required for the formation of three-dimensional cap structures in *P. aeruginosa* biofilms.^257^ Flagella-driven motility is also important for *T. denticola* to form mixed-species biofilms with *P. gingivalis*.^258^ Two small ribonucleic acids that repress the expression of a type IV pilus subunit in *S. sanguinis* ATCC10556 also suppress biofilm formation.^259^ However, twitching motility was not observed in this strain, and it is not yet clear whether twitching motility is important in oral biofilms. However, gliding motility driven by *Capnocytophaga gingivalis* has been shown to contribute to the organization of polymicrobial oral biofilms.^260^ In fact, gliding *C. gingivalis* cells can transport nonmotile bacteria to other areas of the biofilm.

### 4.3 | Signaling and host interactions

The biofilm matrix helps to position microbial cells in close proximity where cell-cell sensing and signaling are optimized.^230^ Signaling between taxonomically distinct bacteria is mediated by autoinducer-2,^261^ whereas signaling between closely related strains of oral *Streptococcus* spp involves peptides, such as competence-stimulating peptide or *S. mutans comX*–inducing peptide.^262^ Extracellular proteases may also be important for cell-cell signaling. For example, the *S. gordonii* serine protease Challisin can interfere with *S. mutans* cell-cell communication by degrading competence-stimulating peptide^263^ or can scavenge amino acids from the surface of *A. oris* cells that are then sensed by *S. gordonii*.^149^

There is evidence that oral biofilms elicit responses in epithelial cells that are distinct from responses to planktonic cells of the same species.^264^ In addition, responses of epithelial cells to multispecies biofilms are different from the sum of the effects of individual species.^265,266^ Many biofilm matrix molecules, including lipopolysaccharides, peptidoglycans, and extracellular DNA, are pathogen-associated molecular patterns that are recognized by immune cells, such as neutrophils and macrophages. However, there is evidence that immune responses are attenuated by bacteria in biofilms. For example, *S. aureus* biofilms have been shown to dampen host immune responses and to induce macrophage dysfunction and cell death.^267^ Similarly, a biofilm-forming strain of *Prevotella intermedia* was shown to resist phagocytosis by polymorphonuclear leukocytes, and a mannose-rich exopolysaccharide was essential for resistance.^268^

### 4.4 | Extracellular pool of nutrients and genes

The matrix provides an important source of extracellular nutrients for bacteria within the biofilm. Macromolecules, such as polysaccharides, proteins, and extracellular DNA, may be broken down into monomers or oligomers that can then be internalized by bacteria. Thus, fructans are thought to serve primarily as extracellular nutrients, since they are produced rapidly by oral bacteria but do not accumulate to high levels in dental plaque.^269^ Further, *P. aeruginosa* has been shown to use extracellular DNA as a source of nutrients.^270^ The biofilm matrix also traps small molecules, including micronutrients such as metal cations. *P. gingivalis* vesicles are highly enriched in proteins IhtB and HmuY, which are involved in the acquisition of heme iron, indicating that vesicles may scavenge and concentrate iron from the host.^222^ Iron has been identified as a growth-rate–limiting nutrient in *P. aeruginosa* biofilms^249^ and may also limit the growth of black-pigmented oral anaerobes, such as *P. gingivalis*, which are highly dependent on iron acquisition for porphyrin pigment production. As well as providing nutrients for biofilm bacteria, the extracellular DNA component of the matrix may serve as a pool of genes for oral bacteria. As noted earlier, transformation was first discovered in *S. pneumoniae*, and many oral streptococci are naturally transformable.^271^ More recently, it has been shown that gram-negative periodontal pathobionts *P. gingivalis* and *T. forsythia* undergo natural transformation.^272,273^ Genes for antimicrobial resistance are common in the oral microbiome, and extracellular DNA–mediated gene transfer has been demonstrated in oral biofilms.^274–276^ In addition, there is evidence from genome sequence analysis that mosaic genes encoding antibiotic-resistant forms of penicillin-binding proteins have arisen through horizontal gene transfer between oral streptococci and *S. pneumoniae*.^277^ In view of the current global antimicrobial resistance crisis, it is critical to elucidate the role of extracellular DNA in horizontal gene transfer and to develop approaches to minimize the spread of genes between bacteria.

## 5 | IMPLICATIONS FOR PERIODONTITIS CONTROL

**FIGURE 7:**
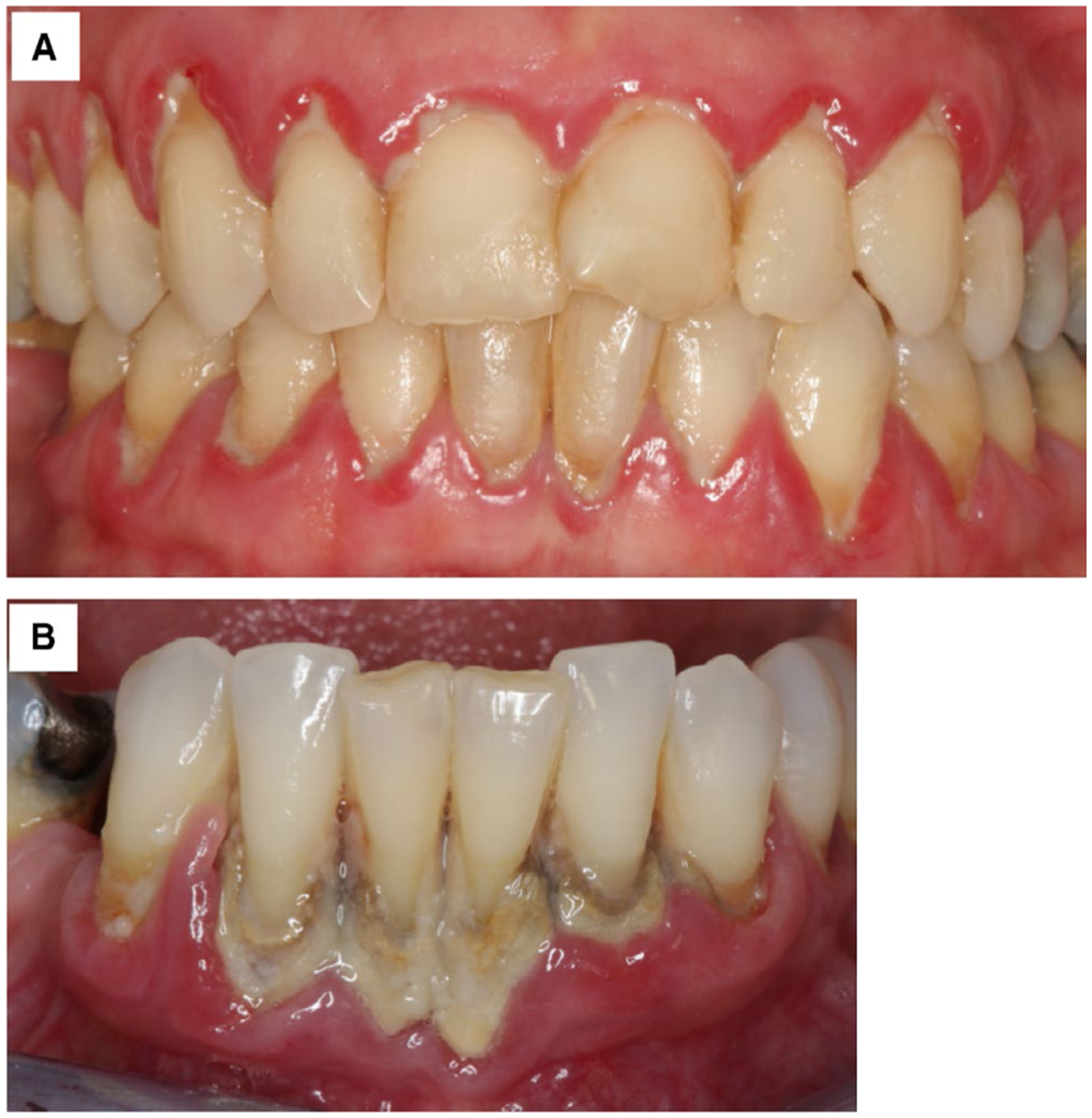
Clinical images of dental plaque. A, A 49-year-old female patient with insufficient oral hygiene, presenting massive amounts of subgingival plaque and clinical signs of plaque-associated gingivitis. B, A 53-year-old female patient with insufficient oral hygiene, presenting massive amounts of subgingival plaque and calculus and clinical signs of advanced periodontitis

Accumulation of subgingival biofilms initiates development of clinical signs of gingivitis (see Figure 7A for a clinical example). As a result of complex interactions between subgingival biofilms and the host immune response, gingivitis can further develop to periodontitis with concomitant loss of periodontal supportive tissues (Figure 7B).^239,278,279^ Periodontal treatment approaches are first and foremost based on complete removal of subgingival biofilms and associated calculus deposits, but they may also comprise adjunctive use of antimicrobials, either applied as local dressings or systemically.^240^ In the following, the implications of our current understanding of the structure and the characteristics of the subgingival biofilm matrix on clinical periodontal treatment are discussed with regard to mechanical removal of subgingival biofilms, as well as to the delivery of antimicrobials to these biofilms.

### 5.1 | Mechanical removal

**FIGURE 8:**
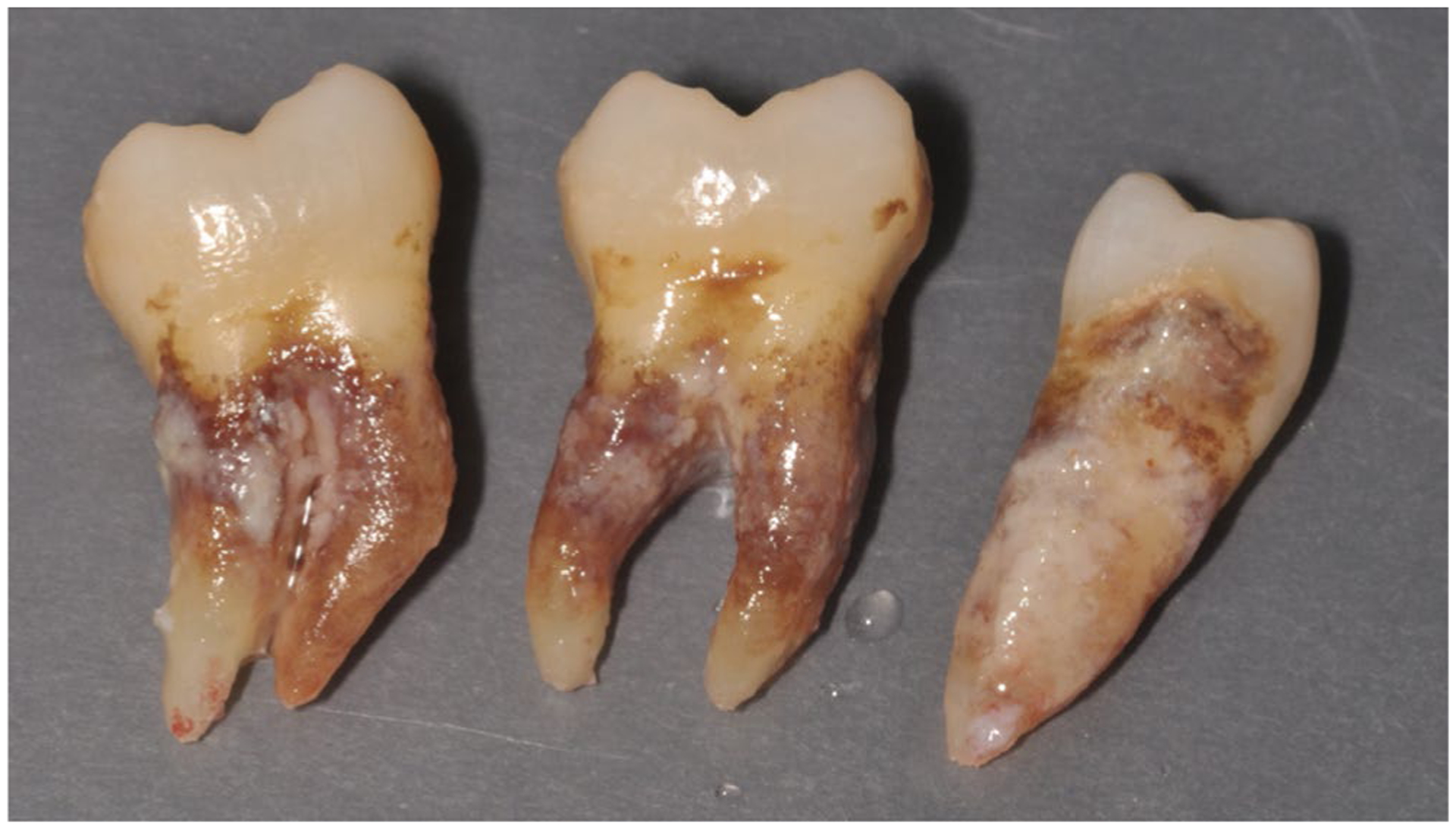
Dental plaque and calculus on the surface of extracted teeth. Teeth 37, 36, and 35 were extracted due to periodontal disease. All teeth present massive amounts of subgingival plaque and calculus

The logical first step in every periodontal treatment is mechanical removal of subgingival biofilms and calculus deposits.^239,280,281^ Although this subgingival debridement most often shows successful clinical outcomes in terms of reductions in probing pocket depth, it still proves to be a tough job in a clinical environment. This is because it is technically demanding and further impeded by limited access and impaired visibility into deeper periodontal pockets.^282^ In a classic study, Rateitschak-Plüss et al^283^ investigated the possibilities and limitations of manual subgingival debridement in 10 single-rooted teeth from four patients by scanning electron microscopy. Eleven out of 40 instrumented root surfaces exhibited residual plaque and “islands” of calculus. These plaque and calculus residues were detected in irregularities of the root surfaces such as fine grooves, ridges, or lacunae, and in areas where the operator may have changed from one curette to another.^283^ Therefore, removal of calculus and plaque in furcation sites of multirooted teeth may be limited,^281^ even when subgingival debridement is aided by endoscopy.^284^ Residual dental plaque and calculus are sometimes clearly visible on teeth extracted for periodontal disease (Figure 8).

Biofilms are generally able to resist mechanical challenges to a certain extent due to the physical protection provided by the biofilm matrix, and in particular by its exopolysaccharide components.^13,234^ Removal of biofilms can therefore only be accomplished by overcoming the cohesive and adhesive forces provided by the biofilm matrix.^13^ When a mature biofilm is subjected to mechanical forces, it shows complex viscoelastic properties, as described earlier.^234,285^ Depending on the strength of the force acting on the biofilm matrix, it will either undergo reversible elastic responses or irreversible deformation. When the detaching forces exceed the cohesive and adhesive forces provided by the biofilm matrix, failure will occur in the biofilm (cohesive failure) or between the substrate and the biofilm (adhesive failure).^234^ This viscoelastic nature of a biofilm may not be a crucial factor when its removal is accomplished by forces applied directly (eg, by means of a curette), but it is of vital importance when hydrodynamic forces are applied in a noncontact mode (eg, by means of sonic scalers during subgingival debridement or by brushing with powered toothbrushes).^234,286,287^ Busscher et al^286^ investigated the noncontact effects of powered toothbrushes on removal of biofilms formed in vitro from *S. oralis* and *A. naeslundii*. They proposed that a biofilm reacts to the absorption of “brush energy” (hydrodynamic force) by viscoelastic expansion of the whole biofilm structure. If enough energy is absorbed and the deformation of the biofilm exceeds a given yield point, biofilm removal will occur. On the other hand, if the absorbed energy is not sufficient, deformation will occur and the biofilm is expanded but not removed.^234,286^ Fabbri et al^288^ documented the formation of ripples and wrinkles and their migration throughout single-species biofilms from *S. mutans*, *S. epidermidis*, and *P. aeruginosa* in a high-shear environment. They further suggested that these may form from so-called Kelvin-Helmholtz instabilities at the interfaces between two fluids and indicate the onset of turbulence in these biofilms.^288^ Further studies on these phenomena are required to improve noncontact removal of biofilms and delivery of antimicrobials throughout their matrices.

### 5.2 | Antimicrobial therapy

Several distinct classes of antimicrobial agents are routinely used in clinics adjunctively to mechanical biofilm removal, both in oral care products for home use and in the course of professional periodontal treatment.^289,290^ Some oral care products comprise antimicrobial agents, such as chlorhexidine, cetylpyridinium chloride, or natural compounds.^290,291^ Chlorhexidine rinses and gels are also routinely applied concomitantly with periodontal treatment; for example, within full-mouth disinfection concepts.^292^ Furthermore, antibiotics can be used adjunctively, either administered systemically in severe cases of periodontitis or locally in persistent or recurrent active periodontal pockets.^289,291,293^ Finally, alternative antimicrobial approaches, such as antimicrobial photodynamic therapy, have been proposed in view of the rising threats from antimicrobial resistance.^291^

There is general consensus that thorough subgingival debridement needs to be performed preceding adjunctive antimicrobial therapy in order to disrupt the subgingival biofilm structure.^289^ This is because bacteria embedded in biofilms can be up to 1000 times more tolerant toward antimicrobials than their planktonic counterparts.^294,295^ For instance, it was found that antibiotics commonly used in periodontics caused reductions only of approximately one log_10_ step in the Zurich biofilm model of subgingival plaque comprising 10 periodontitis-associated bacterial species when applied in concentrations that can typically be reached in gingival crevicular fluid.^296^ Likewise, Wang et al^297^ reported biofilm eradication concentrations of 800 μg/mL metronidazole for an in vitro double-species biofilm of *F. nucleatum* and *P. gingivalis*, whereas minimum bactericidal concentrations for the same bacterial species in planktonic cultures were 25 μg/mL and 10 μg/mL, respectively. This, however, is not a new finding; on the contrary, it was first described as early as 1683 in the famous letter written by Antony van Leeuwenhoek: “From hence I conclude, that the Vinegar with which I washt my Teeth, kill’d only those Animals which were on the outside of the scurf, but did not pass thro the whole substance of it.”^298^ Though it is now understood that the etiology behind this enhanced tolerance of biofilm-embedded bacteria toward antimicrobials is multifactorial,^299^ we will focus our attention in this review on those aspects associated with the biofilm matrix, its structure, or its individual components.

First of all, a given antimicrobial needs to penetrate the biofilm (and its matrix) in order to reach bacteria in the deeper layers of the biofilm. The degree of penetration thereby depends on the thickness and the sorptive capacities of the biofilm, as well as on factors associated with the respective antimicrobial, such as its effective diffusivity within the biofilm, its reactivity with biofilm components, and its concentration and period of application.^244,300^ Consequently, distinct antimicrobial agents will show differential patterns of distribution within a biofilm.^301^ In a comprehensive review,^244^ Stewart combined data from in vitro studies on antimicrobial penetration through biofilms and plotted the penetration times of antimicrobials that had been experimentally measured in in vitro biofilms vs the molecular weight of the respective antimicrobials. He found two groups of agents exhibiting retarded penetration: (a) reactive oxidants, such as chlorine and hydrogen peroxide; and (b) cationic molecules, including some antibiotics, chlorhexidine, or quaternary ammonium compounds (eg, cetylpyridinium chloride). Stewart attributed their limited penetration characteristics to reactions and sorption of the agents with matrix components.^244^ Accordingly, De Beer et al^302^ showed that the limited antimicrobial activity of chlorine in biofilms from *Klebsiella pneumoniae* and *P. aeruginosa* was due to limited penetration stemming from reaction-diffusion interactions. They further suggested that the biofilm matrix with its extracellular polymeric substances may be the substrate for neutralization of chlorine. Tseng et al^303^ found that the positively charged antibiotic tobramycin was sequestered in the outer layers of *P. aeruginosa* biofilms, whereas the neutral antibiotic ciprofloxacin readily penetrated the biofilms. As the penetration of tobramycin could be improved by addition of cations, the authors concluded that the reduced penetration may be due to ionic interactions of tobramycin with negatively charged components of the biofilm matrix. It has also been demonstrated by confocal microscopy that positively charged chlorhexidine only affected the outer layers of bacteria within biofilms formed in situ.^304^ However, not only positive charges, but also hydrophobic interactions involving alkyl chains, such as those in quaternary ammonium compounds, may play a considerable role in binding or retention of antimicrobials during diffusion through biofilms.^243^ Last but not least, the viscoelastic properties of a biofilm may play an important role not only in its mechanical stability, as discussed earlier, but also regarding penetration of antimicrobials throughout its structure due to relaxation-structure-composition relationships.^305^

Another important factor with regard to enhanced tolerance of biofilm-bacteria toward antimicrobials is their inactivation by enzymes that either originate from lysed bacteria owing to antimicrobial exposure or from active secretion by the bacteria in the biofilm via membrane vesicles.^299,306^ In a landmark study, Anderl et al^307^ showed that ampicillin was unable to penetrate *K. pneumoniae* biofilms owing to production of the ampicillin-degrading enzyme β-lactamase, whereas, conversely, ampicillin was able to readily penetrate biofilms formed by a mutant not producing β-lactamase. Interestingly, the biofilms formed by the mutant still showed enhanced tolerance to ampicillin, which clearly demonstrates the multifactorial mechanisms of enhanced tolerance exhibited by bacteria in biofilms.^299,307^

The majority of studies on antimicrobial treatment of biofilms reviewed herein have not been conducted on subgingival biofilms, and not even on biofilms formed by oral bacteria. With the emergence of in vitro models that allow culture of microcosm biofilms resembling complex subgingival microbial communities,^308–311^ it should be a major goal for the future to use these models to investigate the effects of the subgingival biofilm matrix on penetration and inactivation of antimicrobials. For now, however, results from studies on other biofilms can only be extrapolated to subgingival biofilms: The biofilm matrix with its respective structural components protects the embedded bacteria (at least in the inner layers of the biofilm) from penetration of antimicrobials, in particular if they carry positive charge. Furthermore, some agents may be inactivated during their diffusion throughout the biofilm structure. In consequence, this may lead to subinhibitory concentration of antimicrobials in the deeper layers of biofilms, which may pose the risk of inducing drug resistances in bacteria.^280,312,313^

## 6 | FUTURE PERSPECTIVES—TARGETING THE BIOFILM MATRIX?

As discussed already, it is clear that the biofilm matrix is a key factor with regard to the enhanced tolerance of biofilm-embedded bacteria toward mechanical removal, as well as toward antimicrobial approaches. The optimal approach would be to prevent the matrix forming in order to arrest the transition to a pathogenic biofilm state. Inhibition of streptococcal glucosyltransferases has been investigated for many years. Simple sugars, such as maltose, have been shown to inhibit enzyme activity.^314^ More recently, small-molecule inhibitors have been identified that can reduce dental caries in animal models.^315^ Once the matrix has formed, targeting the biofilm matrix and disintegrating its bonds and shielding effects will lead not only to dispersal of biofilms and release of planktonic cells but also to changes in gene expression in bacteria, potentially making them more susceptible toward antimicrobials.^316^

In this regard, matrix-degrading or biofilm-dispersing enzymes have been discussed as potential therapeutic agents.^317^ Among these, Dispersin B and deoxyribonucleases have been the focus of recent research.^301,318,319^ As already noted, Dispersin B is a glycoside hydrolase produced by *A. actinomycetemcomitans*^319^ that has been shown to degrade the glycosidic linkages of polymeric β−1,6-*N*-acetyl-glucosamine (poly-*N*-acetyl-d-glucosamine).^318^ Dispersin B detaches preformed biofilms from *A. actinomycetemcomitans*^320^ and other poly-*N*-acetyl-d-glucosamine–producing bacteria, such as *S. epidermidis*.^321,322^ Recently, it was shown that Dispersin B significantly inhibited colonization of *S. epidermidis* on porcine skin and detached preformed biofilms.^323^ Although a combination of Dispersin B and triclosan has shown successful prevention of *S. aureus* colonization when used as a catheter coating in a rabbit model in vivo,^324^ Dispersin B must still be considered to be in its preclinical stages of development.^325^ In view of the wide range of polysaccharides that contribute to the biofilm matrix, it is likely that matrix degradation would require a combination of glycosyl hydrolases with different specificities.

Since the discovery by Whitchurch et al^110^ that extracellular DNA is an important structural component within the biofilm matrix, the dispersing effects of exogenously applied deoxyribonucleases on bacterial biofilms have been investigated in many in vitro studies.^115,116,326^ Younger biofilms seem to be more prone to dispersal by deoxyribonuclease than more mature ones are, whereas the latter may still be affected by increasing their antimicrobial susceptibility.^115,317,327,328^ Human recombinant deoxyribonuclease has already been employed clinically in the course of treating cystic fibrosis for at least two decades,^111^ and is also emerging as a therapy for bacterial vaginosis.^115,329^ Whereas application of human deoxyribonuclease on a wider scale is still limited by its high costs,^115,317^ bacterially derived deoxyribonucleases, like NucB from *Bacillus licheniformis*, can be produced cost-effectively and have also already shown high potential in vitro.^12^ As matrix-degrading enzymes do not kill bacteria, there should be little or no risk of induction of resistance to these agents.^317^ However, potential immunogenic properties must be ruled out before any clinical application, such as in mouthwashes or toothpastes, for control of subgingival biofilms.^316^

Similarly, antibodies derived against the DNABII family that maintain the structural integrity of the extracellular DNA–dependent extracellular polymeric substances have been shown to disrupt biofilms of multiple bacterial species and have no apparent limitation due to the age of the biofilm.^117^ Likewise, these same antibodies disrupt biofilms without killing the resident bacteria. Importantly, the newly released bacteria are four to eightfold more sensitive to antibiotics than the original planktonic bacteria that seeded the biofilm.^117^ Since anti–DNABII antibodies are effective both in vitro and in vivo on *A. actinomycetemcomitans* periodontal biofilms,^164^ using a cocktail of these antibodies and antimicrobials may be an interesting approach for adjunctive use in clinics.

Other interesting approaches targeting the biofilm matrix include bacteriophages, d-amino acids, modulation of cyclic dimeric guanosine monophosphate signaling pathways, or inhibitors of extracellular metabolic enzymes.^316,317^ Bacteriophages have been shown to play important roles in biofilm development, and particularly in the phase of detachment. Some bacteriophages have been shown to incorporate polysaccharide depolymerase enzymes that can degrade the biofilm matrix of susceptible biofilms, leading to biofilm dispersal.^330,331^ A bacteriophage engineered to express Dispersin B for simultaneously attacking bacterial cells and the biofilm matrix, showed a reduction of viable cell counts of about 4.5 log_10_ units.^332^ Kolodkin-Gal et al^333^ showed that treatment of biofilms formed by *Bacillus subtilis* with d-amino acids (d-leucine, d-methionine, d-tyrosine, d-tryptophan) caused release of amyloid fibers that linked bacterial cells in biofilms together and suggested that production of d-amino acids in biofilms may be a general strategy for biofilm dispersal. This d-amino acid mixture has recently been shown to be effective in dispersing biofilms in dental unit waterlines.^334^ Furthermore, d-leucine could effectively disperse biofilms of *E. faecalis* on human dentine slabs.^335^ Cyclic dimeric guanosine monophosphate is one of the most important bacterial second messengers, playing a key role in the transition from the planktonic to the biofilm lifestyle of bacteria, whereby intracellular levels of cyclic dimeric guanosine monophosphate induce biofilm dispersal.^336^ Therefore, inhibition of diguanylate cyclase, the enzyme synthesizing cyclic dimeric guanosine monophosphate, should promote biofilm dispersal.^317,337^ As the cyclic dimeric guanosine monophosphate signaling system is found in bacteria but not in eukaryotic cells, it may be an attractive target for antibiofilm therapies.^338^ However, the effects of diguanylate cyclase inhibition are not easy to predict, as diguanylate cyclase comprises a large superfamily of enzymes with many homologues in some bacteria.^317^ The protein component of the biofilm matrix includes a number of active enzymes that contribute to pathogenicity. Neuraminidase inhibitors, such as zanamivir, are employed in the treatment of influenza, and they have also shown potential for controlling bacterial pathogens, such as *Gardnerella vaginalis*.^339^ It is possible that the selective inhibition of microbial biofilm matrix enzymes could modulate biofilm functions, such as the ability to stimulate inflammation.

Though biochemical approaches to control oral biofilms are important, it is likely that these will always be an adjunct to mechanical removal approaches. Brushing and flossing are the mainstay of oral hygiene, but they have limited efficacy in difficult-to-reach areas of the teeth. In addition, people with physical disabilities may find manual cleaning measures difficult to use. New approaches designed to overcome these limitations include high-velocity water microdroplets, which can physically remove biofilms in interproximal spaces.^287^ More controlled physical cleaning may eventually be performed by robots. A recent report has described dual-function catalytic antimicrobial robots that are driven by magnets and are highly effective at eradicating biofilms in vitro.^340^ It remains to be seen whether such sophisticated systems can be adapted for controlling dental plaque.

Overall, targeting the biofilm matrix seems to be a very elegant and exciting way to disrupt bacterial biofilms, such as those present in periodontal pockets, and additionally enhance the efficacy of concomitantly applied antimicrobials. Nevertheless, for most of the approaches described herein, research is still in its infancy, and many questions still need to be answered before these agents can be applied clinically. In addition, there are still many unanswered questions regarding the role of the biofilm matrix in the complex interplay between bacteria and host. In multispecies biofilms, the matrix will consist of many different classes of polymers, and numerous variants of each. Therefore, simplified models of oral biofilms are still critical to further unravel the structure and function of biofilm matrix components.
